# Safety and immunogenicity of a reduced dose of the BNT162b2 mRNA COVID-19 vaccine (REDU-VAC): A single blind, randomized, non-inferiority trial

**DOI:** 10.1371/journal.pgph.0001308

**Published:** 2022-12-20

**Authors:** Pieter Pannus, Stéphanie Depickère, Delphine Kemlin, Sarah Houben, Kristof Y. Neven, Leo Heyndrickx, Johan Michiels, Elisabeth Willems, Stéphane De Craeye, Antoine Francotte, Félicie Chaumont, Véronique Olislagers, Alexandra Waegemans, Mathieu Verbrugghe, Marie-Noëlle Schmickler, Steven Van Gucht, Katelijne Dierick, Arnaud Marchant, Isabelle Desombere, Kevin K. Ariën, Maria E. Goossens

**Affiliations:** 1 Scientific Direction Infectious Diseases in Humans, Sciensano, Brussels, Belgium; 2 Institute for Medical Immunology and ULB Centre for Research in Immunology (U-CRI), Université Libre de Bruxelles (ULB), Gosselies, Belgium; 3 Department of Biomedical Sciences, Virology Unit, Institute of Tropical Medicine, Antwerp, Belgium; 4 Mensura EDPB, Occupational Health Service, Antwerp, Belgium; 5 Department of Biomedical Sciences, University of Antwerp, Antwerp, Belgium; University of Oslo Faculty of Medicine: Universitetet i Oslo Det medisinske fakultet, NORWAY

## Abstract

Fractional dosing of COVID-19 vaccines could accelerate vaccination rates in low-income countries. Dose-finding studies of the mRNA vaccine BNT162b2 (Pfizer-BioNTech) suggest that a fractional dose induces comparable antibody responses to the full dose in people <55 years. Here, we report the safety and immunogenicity of a fractional dose regimen of the BNT162b2 vaccine. REDU-VAC is a participant-blinded, randomised, phase 4, non-inferiority study. Adults 18–55 years old, either previously infected or infection naïve, were randomly assigned to receive 20μg/20μg (fractional dose) or 30μg/30μg (full dose) of BNT162b2. The primary endpoint was the geometric mean ratio (GMR) of SARS-CoV-2 anti-RBD IgG titres at 28 days post second dose between the reduced and full dose regimens. The reduced dose was considered non-inferior to the full dose if the lower limit of the two-sided 95% CI of the GMR was >0.67. Primary analysis was done on the per-protocol population, including infection naïve participants only. 145 participants were enrolled and randomized, were mostly female (69.5%), of European origin (95%), with a mean age of 40.4 years (SD 7.9). At 28 days post second dose, the geometric mean titre (GMT) of anti-RBD IgG of the reduced dose regimen (1,705 BAU/mL) was not non-inferior to the full dose regimen (2,387 BAU/mL), with a GMR of 0.714 (two-sided 95% CI 0.540–0.944). No serious adverse events occurred. While non-inferiority of the reduced dose regimen was not demonstrated, the anti-RBD IgG titre was only moderately lower than that of the full dose regimen and, importantly, still markedly higher than the reported antibody response to the licensed adenoviral vector vaccines. These data suggest that reduced doses of the BNT162b2 mRNA vaccine may offer additional benefit as compared to the vaccines currently in use in most low and middle-income countries, warranting larger immunogenicity and effectiveness trials.

**Trial Registration:** The trial is registered at ClinicalTrials.gov (NCT04852861).

## Introduction

Today, less than 22% of people in low-income countries (LIC) have been vaccinated against COVID-19 with at least one dose, compared to 72% in high-income countries [1]. Besides allowing the emergence of new SARS-CoV-2 variants and their subsequent spread around the world, this blatant inequity in global vaccine distribution is leaving hundreds of millions of people vulnerable to the deadly virus. According to the UN, vaccine inequity will further deepen inequality and have a lasting impact on socio-economic recovery in LIC [2].

The lack of access to COVID-19 vaccines is partly due to a phenomenon called ‘vaccine nationalism’, wherein most vaccines are being reserved for wealthy countries, and vaccine-producing countries limit exports to make sure that their own population is vaccinated first. Another important factor is the cost. Under current pricing, LIC would have to increase health care expenditure by up to 50%, compared to less than 1% for high-income countries, to vaccinate 70% of their population [3–5]. The WHO, Gavi and CEPI co-led program COVAX was set up to stimulate global equitable access to COVID-19 vaccines. Although this program has led to the donation of over 1 billion vaccine doses to LIC to date, more doses, better logistics, and much more political will are required to cover the needs of the Global South [6].

Fractionating doses as a dose-sparing strategy might help speed up vaccination rates by effectively reducing the cost per dose and thereby increasing the number of available doses. Based on proven safety and immunogenicity, the World Health Organization (WHO) has recommended fractional doses in the past during outbreaks of yellow fever, polio and meningococcal disease in case of vaccine shortages in resource-limited settings [7–10].

In terms of fractional vaccine doses for COVID-19 however, data is mostly limited to dose-finding studies. In a phase 1/2 trial in healthy adults aged 18–55 years, humoral and cellular responses to the BNT162b2 mRNA vaccine (Comirnaty, Pfizer-BioNTech) were found to be very similar between the full dose of 30μg and fractional doses of 20μg and even 10μg [11]. For the mRNA-1273 vaccine (Spikevax, Moderna), a phase 2 trial showed comparable humoral responses (binding and neutralizing antibody titres) to a 50μg dose as compared to the full dose of 100μg [12]. As far as we know, there has only been one trial reporting on clinical efficacy of fractional dosing in a small number of subjects. This multicentre trial observed 67%-97% efficacy of protection against symptomatic COVID-19 in a sub-group of participants who were primed with a half dose and boosted with a full dose of the ChAdOx1 nCoV-19 (Vaxzevria, AstraZeneca) vaccine [13]. In a recent paper, Więcek *et al*. used modelling data by Khoury *et al*. to predict vaccine efficacy of fractional doses [14,15]. Compared to the 95% efficacy provided by the full dose of 30μg, fractional doses of 10μg and 20μg of BNT162b2 are estimated to still be 80–90% effective in adults below 55 years old. Together with the dose-sparing advantage, the possibly fewer side effects caused by lower dosing, as suggested by some clinical data, may help tip the balance in favour of implementing fractional dosing strategies [16,17].

As more data is urgently needed to fill this knowledge gap and guide the potential use of fractional doses of mRNA vaccines, we conducted a randomised controlled trial to determine whether immune responses to a reduced dose of BNT162b2 (20μg) are non-inferior to the full, licensed dose (30μg). We found that binding and neutralizing antibody responses to the reduced dose of 20μg were indeed inferior to the full dose of 30μg of BNT162b2, but nevertheless still markedly higher than responses induced by other approved COVID-19 vaccines with proven efficacy.

## Methods

### Study design

REDU-VAC is a participant-blinded, randomised, phase 4, multicentre, non-inferiority study investigating safety, reactogenicity and immunogenicity of a fractional dose of the mRNA COVID-19 vaccine BNT162b2 (Pfizer-BioNTech). Recruitment occurred at five sites across Belgium of the ‘External Department of Prevention and Protection at work’ of Mensura.

Two prime-boost schedules are compared consisting, respectively, of two doses of 20μg (reduced dose schedule) and two doses of 30μg (full dose schedule) of BNT162b2. All participants were randomly assigned to receive either the reduced or the full dose schedule.

### Ethics and registration

The trial was reviewed and approved by the Erasme Hospital Ethics Committee (P2021/251) and the Federal Agency for Medicines and Health Products (EudraCT: 2021-002088-23A). The trial was registered at ClinicalTrials.gov (NCT04852861). The protocol is available in S1 Appendix, the CONSORT checklist in S2 Appendix.

### Participants

Non-previously vaccinated adults aged 18–55 years old who were in good general health or having well-controlled co-morbidities, either infection naïve or previously infected, were eligible. Exclusion criteria included history of severe adverse reactions associated with a vaccine or severe allergic reaction to any component of the study intervention, acute severe febrile illness or acute infection, pregnancy and breastfeeding.

### Randomisation and masking

Participants were block-randomised (random block size between 2, 4, 6, and 8, ratio of 1:1:1:1) within female/20μg, female/30μg, male/20μg, and male/30μg using the blockrand R package by the study statistician [18]. The age distribution in each group was then verified to be not different in the four groups using a Kruskal-Wallis test. The obtained p-value was 0.54.

Participants and laboratory staff were masked to the administered vaccine dose (20μg versus 30μg). To ensure participant blinding to the vaccine dose, randomisation lists were kept out of sight, vaccines were prepared, and syringes were filled beforehand.

### Procedures

Participants were informed of the study and asked to register online before study participation. Those meeting all inclusion criteria were randomly assigned to one of the two study groups by the study statistician and were invited to the baseline visit (day 0) by the study nurses. At the baseline visit, participants provided written informed consent before having blood drawn and being vaccinated.

Two different dosages of the same COVID-19 mRNA BNT162b2 vaccine were used in the study. BNT162b2 is a lipid nanoparticle–formulated, nucleoside-modified mRNA vaccine encoding a prefusion stabilized, membrane-anchored SARS-CoV-2 full-length spike protein. The 20μg and 30μg doses were administered via respectively a 0.2 mL and a 0.3 mL intra-muscular injection into the upper arm.

Vaccines were administered by trained study nurses at the different trial sites. Participants were observed for a minimum of 15 minutes after vaccination. The interval between the two vaccine doses was three weeks for all participants. Blood was drawn on days of vaccination (day 0 and day 21), four weeks post second dose (day 49), and six months post first dose (month six). One week after administration of each vaccine dose, participants were asked to record online any experienced local and systemic adverse events as well as their severity (mild/moderate/severe). Participants were also asked to record breakthrough infections by registering online the date of a positive SARS-CoV-2 molecular test (on the condition that no third vaccine dose had been received yet), and in case of symptomatic infection, the duration of symptoms. Breakthrough infections were followed-up until administration of a third vaccine dose.

SARS-CoV-2 anti-receptor binding domain (RBD) specific IgG concentrations were measured by ELISA (reported as Binding Antibody Units [BAU]/mL) on days 0/21/49 and month 6. Neutralizing antibody titres against SARS-CoV-2 Wuhan (2019-nCoV-Italy-INMI1, reference 169 008V-03893) (days 21/49), the B.1.617.2 Delta variant (83DJ-1) and the BA.1 Omicron variant (day 49) were measured with a live virus neutralization assay (VNA, reported as 50% neutralization, NT50) [19]. The VNA was only performed against Delta and Omicron for samples with an NT50 (Wuhan) titre >50 and >400 respectively. Cellular responses were measured at day 49 both with IFN-γ enzyme-linked immunosorbent spot assay (ELISpot) as well as with multi-colour flow cytometry on a subsample of 45 randomly selected participants. Detailed methods are described in the S2 Appendix.

Previous infection status was established following a decision tree (S1 Fig). Participants with a previous laboratory-confirmed SARS-CoV-2 infection were considered previously infected irrespective of baseline serology. All other participants with a baseline anti-RBD IgG titre <5 BAU/mL were considered infection naïve, and those ≥ 30 BAU/mL were considered previously infected. Participants with a titre ≥ 5 and < 30 BAU/mL were further tested with a different, multiplexed immunoassay (Multi-SARS-CoV-2 Immunoassay) detecting four targets: RBD, spike subunit 1 (S1), spike subunit 2 (S2) and nucleocapsid (N) [19]. Participants with ≥3 out of 4 targets positive were considered previously infected.

### Outcomes

The primary outcome was serum SARS-CoV-2 anti-RBD IgG concentration 28 days post second dose (day 49) in infection naïve participants. Secondary outcomes included reactogenicity, as measured by reported local and systemic adverse events in the week following each vaccination, and safety, as measured by suspected unexpected serious adverse reactions, serious adverse reactions, and adverse reactions with grade equal or more than three over the entire study period.

Immunological secondary outcomes include SARS-CoV-2 anti-RBD IgG at day 0, day 21 and month 6; VNA titres at days 21 (Wuhan) and 49 (Wuhan, Delta and Omicron); and SARS-CoV-2 specific cell frequencies at day 49 in infection naïve participants only as well as in infection naïve and previously infected participants combined.

### Statistical analysis

The sample size was calculated assuming a true difference of geometric means of the primary outcome on the log_10_ scale being 0 between the reduced and the full dose, and a standard deviation of GMT on the log_10_ scale being 0.27 [19]. A minimum of 50 naïve participants per arm was necessary to achieve 90% power at a two-sided 5% significance level. The geometric mean ratio (GMR) was then calculated as the anti-logarithm of the difference between the mean on the log_10_ scale of the primary outcome in the reduced dose and that in the full dose (reference dose), after adjusting by a mixed-effect linear model using age, gender, and baseline titre of SARS-CoV-2 anti-RBD IgG as fixed factors and study sites as random factor. To conclude on non-inferiority between the two groups, the WHO criterion of 0.67 was used [20], i.e. the reduced dose was considered non-inferior when the lower limit of the two-sided 95% CI of the GMR was greater than this cut-off. In the same way, GMRs were also calculated at day 49 for VNA titres against Wuhan and Delta variants.

The proportion of participants with responses higher than the lower limit of detection were calculated for SARS-CoV-2 anti-RBD IgG at days 0, 21 and 49, and for VNA titres at days 21 (Wuhan) and 49 (Wuhan, Delta, Omicron) with 95% CI calculated by the binomial exact method. They were compared between the reduced and the full dose groups using the Fisher’s exact test. Data below the lower limit of detection were given a value equal to half of the threshold before transformation. Comparisons of primary and secondary outcomes were evaluated by linear mixed-effect model adjusting for age, gender, and baseline titre of SARS-CoV-2 anti-RBD IgG (for days 21 and 49) or naïve/not naïve category (for day 0) as fixed factors and study sites as random factor. Correlations between SARS-CoV-2 anti-RBD IgG and VNA titres (Wuhan), and between VNA against Wuhan and Delta variants were evaluated by Pearson correlation coefficients. Concerning cellular responses, the proportion of participants with responses higher than 0 in IFN-γ ELISpot and with a positive response in flow cytometry was computed at day 49 with 95% CI calculated by the binomial exact method. Fisher’s exact test was used to compare reduced and full dose groups. For IFN-γ ELISpot analysis, data equal to 0 were given a value of 1 before transformation, and for flow cytometry, negative frequencies were given a value of 0.0001 before transformation. Comparisons of GMTs were evaluated by linear mixed-effect model adjusting for age, gender, and baseline titre of SARS-CoV-2 anti-RBD IgG as fixed factors and study sites as random factor. All statistical analyses were done using R version 4.1.2.

## Results

**Fig 1:**
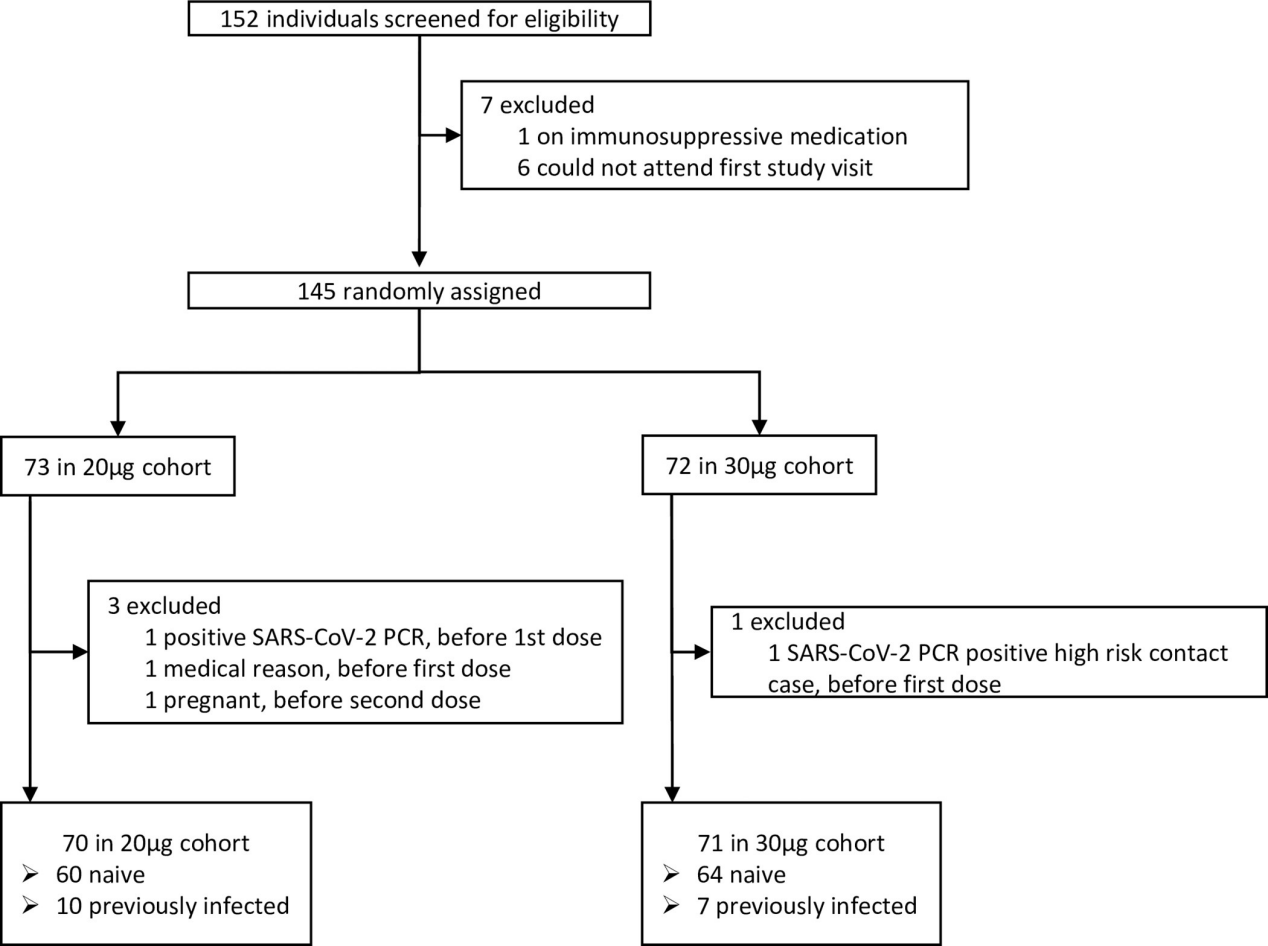
Trial profile.

Between April 19 and April 23, 2021, 152 participants were screened for eligibility in five sites across Belgium among whom 145 were enrolled in the study and randomized (Fig 1). Four participants were excluded from the analysis due to pregnancy, active infection, high-risk contact or medical reasons. Participants were mostly female (69.5%), of European origin (95%), with a mean age of 40.4 years (SD 7.9). Six participants (4.3%) were taking medication to treat a co-morbidity. Baseline characteristics were well balanced across the two study arms (Table 1). Hundred twenty-four participants were considered SARS-CoV-2 infection naïve and 17 were previously infected at baseline. Here, we focus on the results of the per-protocol (PP) analysis including infection naïve participants only and present the results of the intention-to-treat (ITT) analysis, including both naïve and previously infected participants, in supplementary data.

**Table 1:** Baseline characteristics by cohort and study arm.

	Per-protocol		Intention-to-treat	
	20 μg	30 μg	20 μg	30 μg
Participants, n	60	64	70	71
Age, years				
Mean (SD)	40.4 (7.5)	41.0 (8.2)	39.8 (7.9)	41.0 (8.0)
Median (range)	39.5 (23.0–54.0)	41.5 (24.0–55.0)	39.0 (23.0–54.0)	41.0 (24.0–55.0)
Sex				
Female	43 (71.7%)	43 (67.2%)	49 (70.0%)	49 (69.0%)
Male	17 (28.3%)	21 (32.8%)	21 (30.0%)	22 (31.0%)
Ethnicity				
European	56 (93.3%)	63 (98.4%)	65 (92.9%)	69 (97.2%)
Latin	1 (1.7%)	0	1 (1.4%)	0
Asian	1 (1.7%)	0	1 (1.4%)	1 (1.4%)
Sub-Saharan Africa	2 (3.3%)	0	2 (2.9%)	0
Northern-Africa	0	1 (1.6%)	1 (1.4%)	1 (1.4%)
Comorbidities				
Cardiovascular	1 (1.7%)	2 (3.1%)	2 (2.9%)	2 (2.8%)
Oncological	0	1 (1.6%)	0	1 (1.4%)
Respiratory	0	1 (1.6%)	0	1 (1.4%)
BMI				
<18.5	1 (1.7%)	1 (1.6%)	1 (1.4%)	1 (1.4%)
≥18.5 and <25	39 (65.0%)	36 (56.3%)	45 (64.3%)	40 (56.3%)
≥25	20 (33.3%)	27 (42.1%)	24 (34.3%)	30 (42.3%)

Data are n (%) unless otherwise indicated.

**Fig 2:**
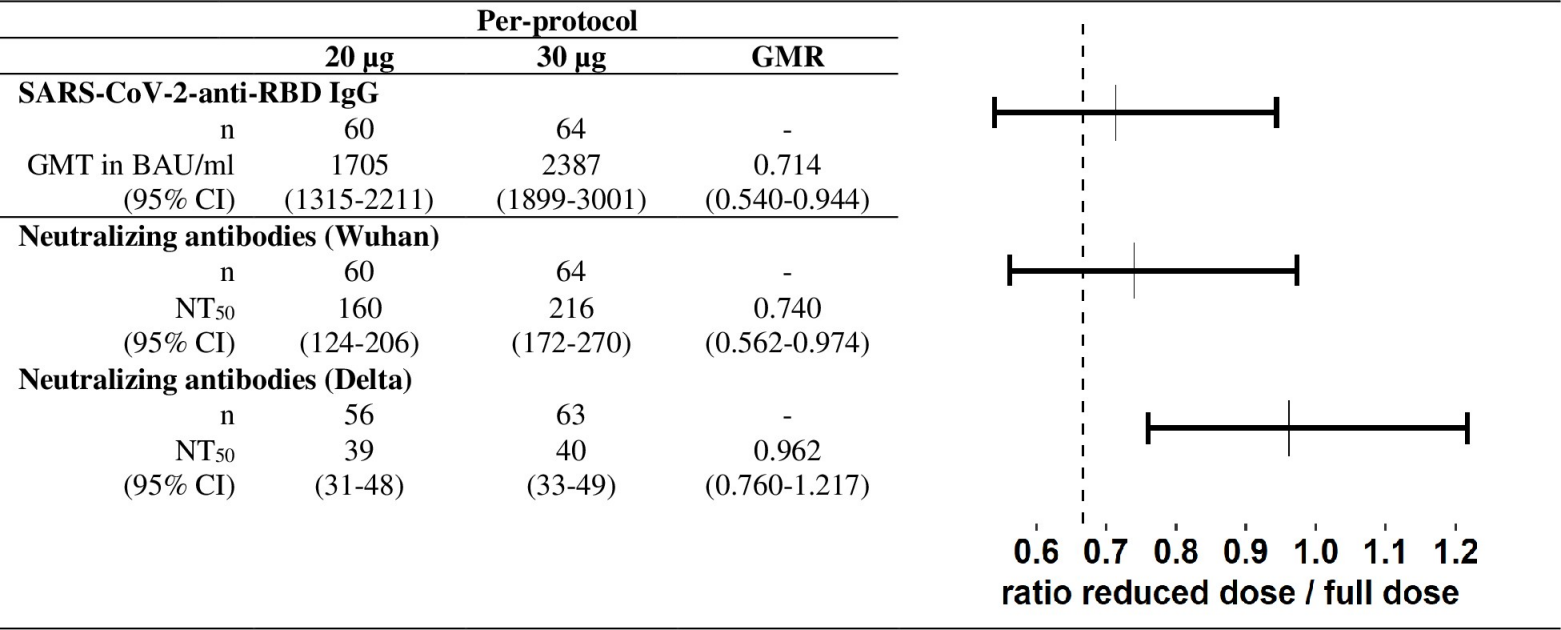
Immune responses by study arm at 28 days post second vaccine dose (Day 49) and non-inferiority analysis in the per-protocol cohort. Data are geometric mean titres (95% CI) at day 28 post second dose. GMRs (95% CI) were adjusted with a linear mixed-effect model including gender, age and SARS-CoV-2 anti-RBD IgG titre at baseline as fixed variables and location as random variable. In the figure, the dashed line indicates the WHO recommended non-inferiority margin of 0.67. GMT = geometric mean titre. GMR = geometric mean ratio. BAU = Binding antibody units. NT50 = 50% neutralizing antibody titre.

At the primary endpoint, 28 days post second dose, the GMT of SARS-CoV-2 anti-RBD IgG in the PP cohort was 1705 BAU/ml in the 20μg group and 2387 BAU/ml in the 30μg group (Fig 2). The GMR of 0.714 (0.540–0.944, 95% CI) indicated that non-inferiority was not demonstrated given that the lower limit of the 95% CI was inferior to the WHO recommended margin of 0.67. Similarly, the ITT analysis did not demonstrate non-inferiority of the reduced dose either, with GMTs of 1822 BAU/ml and 2381 BAU/ml in the 20μg and 30μg arms, respectively, and a GMR of 0.765 (0.593–0.987, 95% CI) (S1 Table).

**Fig 3:**
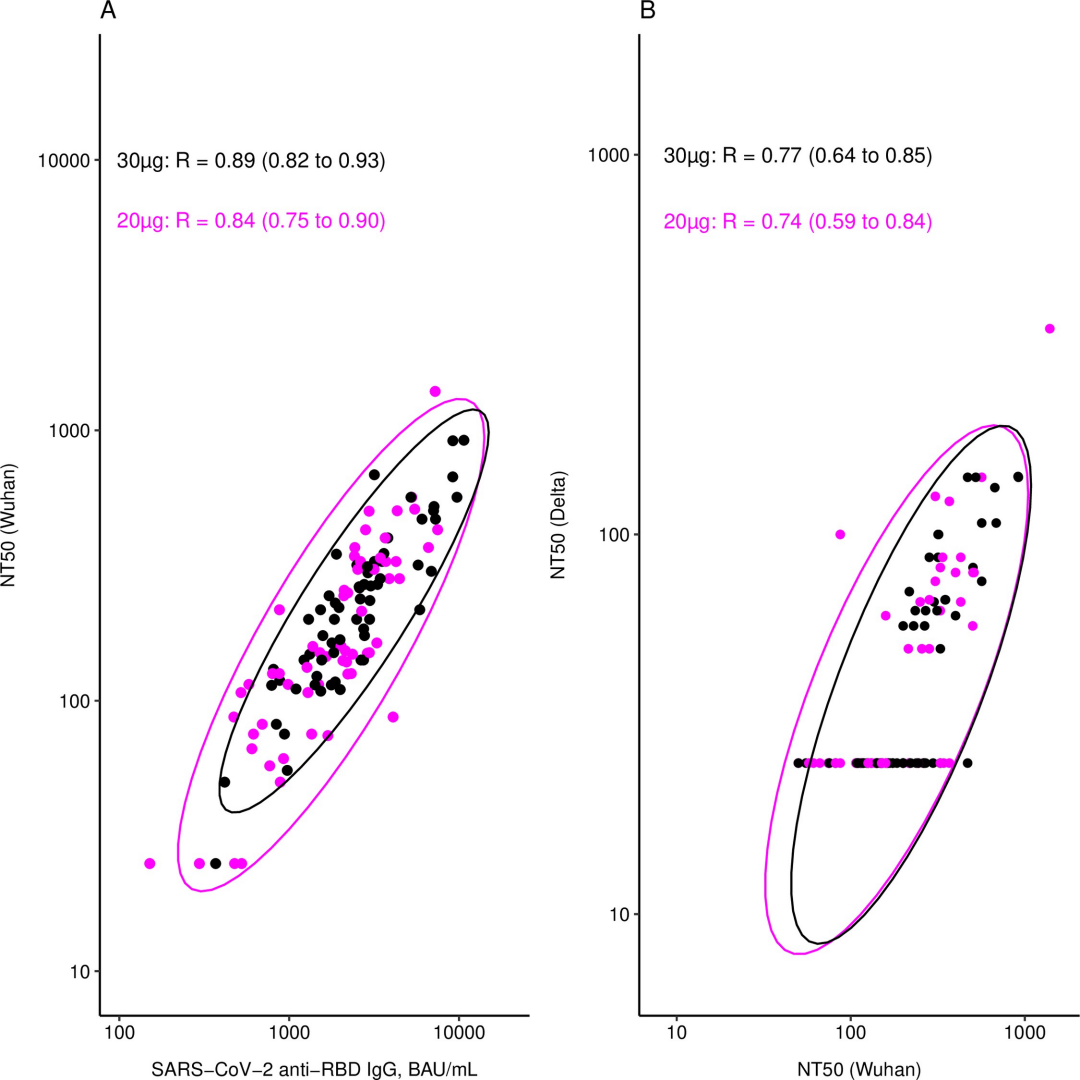
Correlations between immune responses per study arm. Correlations were analysed at 28 days after the second vaccine dose between SARS-CoV-2 anti-RBD IgG binding antibodies and SARS-CoV-2 Wuhan neutralizing antibodies (A), and between SARS-CoV-2 Wuhan and Delta variant neutralizing antibodies (B). Pearson correlation coefficients (95% CI) are given per study arm. Ellipses represent the 95% CI for the two study arms (purple = 20μg, black = 30μg), assuming multivariate normal distributions. NT50 = 50% neutralizing antibody titre, RBD = SARS-CoV-2 receptor binding domain, BAU = binding antibody units.

We observed strong correlations between SARS-CoV-2 binding (anti-RBD IgG) and neutralizing (Wuhan NT_50_) antibody titres in both the reduced and the full dose arms (Fig 3A). As a result, GMRs for neutralizing titres were similar to those described above for binding titres. Indeed, GMTs of neutralizing antibody titres against Wuhan were 160 (124–206) and 216 (172–270) in the 20μg and 30μg arms, respectively, with a GMR of 0.740 (0.562–0.974) (Fig 2). In the ITT analysis, GMTs were 216 (95% CI: 170–276) and 279 (224–347) in the 20μg and 30μg arms, respectively, with a GMR of 0.776 (0.592–1.017) (S1 Table). In both analyses, non-inferiority was not demonstrated.

Neutralizing antibodies against Delta and Omicron were only tested for participants with an NT50 (Wuhan) >50 and >400, respectively. As expected, neutralizing capacity against these variants was much lower compared to Wuhan. In the samples tested, just 21/56 (38%) and 25/63 (40%) naïve participants had detectable Delta neutralizing titres in the 20μg and 30μg arms, respectively (Table 2). For Omicron, none of the naïve participants (0/8 and 0/11 for 20μg and 30μg, respectively) had detectable neutralizing activity. Titres against Wuhan and Delta were strongly correlated in both study arms (Fig 3B). Correlation between Wuhan and Omicron could not be determined due to the mostly undetectable titres. Non-inferiority of the reduced dose to the full dose was demonstrated for Delta neutralizing titres, in the PP and ITT analyses, as the lower limit of the 95% CIs were superior to the WHO margin of 0.67 (Fig 2 and S1 Table). As all Omicron neutralizing titres were undetectable in naïve participants, regardless of the study arm, a non-inferiority analysis was not performed.

**Table 2:** Humoral and cellular responses by study arm in the per-protocol cohort at the different time points.

	Per-protocol		
	20 μg	30 μg	p-value
**SARS-CoV-2 anti-RBD IgG**			
**Day 0**			
n	60	64	
Concentration (BAU/ml)	2.6 (2.3–2.9)	2.8 (2.5–3.0)	p = 0.421
> 5 BAU/ml	2 (3%, 0.4–12)	4 (6%, 1.7–15)	p = 0.681
**Day 21**			
n	60	64	
Concentration (BAU/ml)	117 (84–162)	223 (166–298)	p<0.001
> 5 BAU/ml	59 (98%, 91–100)	64 (100%, 94–100)	p = 0.484
**Day 49**			
n	60	64	
Concentration (BAU/ml)	1705 (1315–2211)	2387 (1899–3001)	p = 0.019
> 5 BAU/ml	60 (100%, 94–100)	64 (100%, 94–100)	p = 1.000
**Month 6**			
n	50	60	p = 0.007
Concentration (BAU/ml)	157 (113–217)	246 (188–323)	
> 5 BAU/ml	50 (100%, 93–100)	60 (100%, 94–100)	p = 1.000
**Neutralizing antibodies**			
**Wuhan—Day 21**			
n	60	64	
NT_50_	27 (24–31)	29 (26–33)	p = 0.355
> 50	7 (12%, 5–23)	10 (16%, 8–27)	p = 0.606
**Wuhan—Day 49**			
n	60	64	-
NT_50_	160 (124–206)	216 (172–270)	p = 0.032
> 50	56 (93%, 84–98)	63 (98%, 92–100)	p = 1.000
**Delta—Day 49**			
n	56	63	
NT_50_	39 (31–48)	40 (33–49)	p = 0.743
> 50	21 (38%, 25–51)	25 (40%, 28–53)	p = 0.852
**Omicron—Day 49**			
n	8	11	
NT_50_	25 (25–25)	25 (25–25)	-
> 50	0 (0%, 0–37)	0 (0%, 0–28)	p = 1.000
**IFN-γ producing cells per million PBMCs**			
**SARS-CoV-2 S1-specific—Day 49**			
n	18	24	
Mean number of cells/million	71.5 (33.0–155.1)	105.9 (60.0–187.0)	p = 0.327
> LOD	12 (67%, 41–87)	19 (79%, 58–93)	p = 0.812
**SARS-CoV-2 S2-specific—Day 49**			
n	18	24	
Mean number of cells/million	88.5 (34.0–230.3)	118.3 (50.9–274.8)	p = 0.509
> LOD	11 (61%, 36–83)	16 (67%, 45–84)	p = 1.000

Data are geometric means (95% CI) for continuous variables, and n (%, 95% CI) for binary values. The LLOQ for the SARS-CoV-2 anti-RBD IgG was 5 BAU/mL, NT50 = 50 for the live virus neutralisation assay, and 54 and 66 cells/million PBMCs for S1 and S2, respectively, for ELIspot. For continuous variables, p-values are reported using a linear mixed-effect model adjusted for gender, age and baseline infection status (for day 0 data) or baseline SARS-CoV-2 anti-RBD IgG titre (for day 21/49 and month 6 data) as fixed variables and location as random variable. Fisher’s exact test was used to report p-values for binary variables. BAU = Binding antibody units. NT50 = 50% neutralizing antibody titre. PBMC = peripheral blood mononuclear cell. S1 = SARS-CoV-2 spike protein subunit 1. S2 = SARS-CoV-2 spike protein subunit 2.

**Fig 4:**
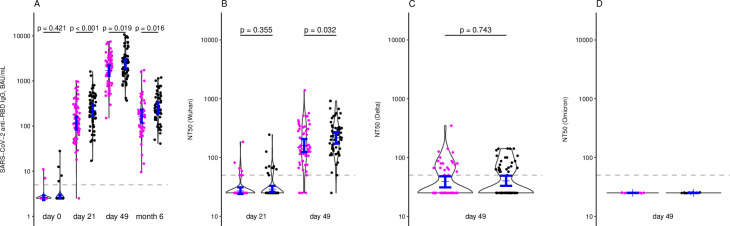
Kinetics of immune responses per study arm of the per-protocol cohort. SARS-CoV-2 anti-RBD IgG binding antibody data are shown on days 0/21/49 and month 6 post first dose (A), SARS-CoV-2 Wuhan neutralizing antibodies on days 21/49 (B) and SARS-CoV-2 Delta (C) and Omicron (D) neutralizing antibodies on day 49 per study arm (purple = 20μg, black = 30μg). In the case of SARS-CoV-2 anti-RBD IgG binding antibody at month 6, five samples were excluded due to a detected breakthrough infection before month 6. Blue bars indicate geometric mean titres with 95% CI. NT50 = 50% neutralizing antibody titre, RBD = SARS-CoV-2 receptor binding domain, BAU = binding antibody units.

In both the reduced and full dose study arms, all participants had seroconverted by 28 days post second dose (Table 2, Fig 4A). However, the GMTs of anti-RBD IgG were significantly lower in the 20μg arm compared to the 30μg arm at the time of the second dose administration (day 21) and 28 days later (day 49). At six months post first dose anti-RBD IgG titres had waned significantly, but all participants remained seropositive. The GMT of anti-RBD IgG was still significantly lower in the reduced dose arm at this time point (Table 2, Fig 4A). In terms of neutralizing activity, 56/60 (93%) versus 63/64 (98%) of naïve participants from the 20μg arm and the 30μg arm, respectively, had detectable neutralizing antibody titres against Wuhan, with a significantly lower titre in the reduced dose versus full dose arm 28 days post second dose (Table 2, Fig 4B). Neutralizing antibodies against Delta and Omicron were only measured at day 49 and did not differ significantly between the two study arms (Table 2, Fig 4C and 4D).

Cellular responses elicited against a pool of SARS-CoV-2 spike protein subunit 1 (S1) and 2 (S2) peptides, measured with IFN-γ ELISpot, were not significantly different either between the reduced and full dose arms, either in the PP or the ITT cohort (Tables 2 and S2). The proportion of participants with detectable responses was also not significantly different, with 67% (41–87) versus 79% (58–93) for S1 in the 20μg and 30 μg arms, respectively, and 61% (36–83) versus 67% (45–84) for S2 in the 20μg and 30 μg arms, respectively (Table 2), in the PP cohort. Similar findings were observed in the ITT cohort (S2 Table). These data were corroborated by flow cytometry, where no differences were observed between the reduced and full dose arms in the proportion of S1 or S2 specific CD4+ and CD8+ T cells expressing CD154 (CD4+ T cells only), IFN-γ, IL-2, and TNF-α (S2 Fig).

A total of 18 breakthrough infections (BTI) were reported and all occurred between five to eight months after the first dose, coinciding with a major infection wave in the country due to the Delta variant (S3 Table). These BTIs occurred exclusively in infection naïve participants and were as likely to occur in the 20μg arm as in the 30μg arm (Fisher’s exact test, p = 0.80). GMTs of binding and neutralizing antibodies at 28 days post second dose or six months post first dose were not significantly different between those experiencing and not experiencing a BTI (Wilcoxon rank sum test, p>0.14).

No serious adverse events were reported during the study period. No difference in frequency or severity of adverse events reported after each vaccine dose was observed between the 20μg and the 30μg arm (S4 Table, S3 Fig). The only exception was the reported severity of nausea after the second dose which was mostly moderate in the 20μg arm and mild in the 30μg arm.

## Discussion

The study results show that the administration of a reduced dose (2x20μg) of the mRNA vaccine BNT162b2 induces lower titres of SARS-CoV-2 anti-RBD IgG binding and SARS-CoV-2 Wuhan neutralizing antibodies than the administration of the full vaccine dose (2x30μg). No such difference was observed for SARS-CoV-2 Delta neutralizing antibodies but this can likely be explained by the overall lower titres as compared to Wuhan, with many participants having undetectable neutralizing antibody titres post vaccination. For Omicron neutralization, none of the naïve participants had detectable titres, precluding a proper comparison between study arms but highlighting the lack of induction of neutralizing capacity against the Omicron variant with two doses of BNT162b2. Our findings contrast those from the phase-I/II trial report by Sahin *et al*. where a double 20μg dose did not induce lower humoral responses than the double full 30μg dose. It is possible that the much larger sample size of our study (70 versus 12 per group) enabled us to reveal the relatively modest difference in response between both study arms.

The ratios of the GMTs across all analyses indicate that the investigated reduced dose regimen induced around 25–30% lower antibody titres than the licensed full dose regimen. The magnitude of this reduction and its potential impact on vaccine efficacy needs to be put into perspective, however. A recent non-inferiority trial compared immunogenicity of Pfizer-BioNTech’s BNT162b2 with Astra-Zeneca’s adenoviral ChadOx1 nCoV-19 vaccine, amongst other vaccination regimens. At 28 days post second dose, the GMT of anti-spike binding IgGs was 90% lower for ChadOx1 nCoV-19 than for BNT162b2 [21]. Despite ChadOx1 nCoV-19’s markedly lower humoral responses, it still has been shown to have an efficacy against symptomatic infection of about 70%, and it has been approved for use in 182 countries with more than two billion doses administered, primarily in LIC [13,22]. While it is possible that other, antibody independent factors contribute to the success of the ChAdOx1 vaccine, the well-documented and strong correlation between vaccine efficacy and neutralizing antibody titres makes us argue that the moderately lower humoral response of the reduced dose regimen investigated in our study is still excellent, and likely provides significant protection against COVID-19 with additional benefit as compared to the non-mRNA vaccines currently in use in most low and middle-income countries [14,15]. In order to support this notion, larger immunogenicity and effectiveness trials are warranted, as well as non-inferiority trials directly comparing lower dosage of e.g. an mRNA vaccine with the standard dosage of an adenoviral vectored vaccine as currently used in low and middle-income countries.

In contrast to the humoral responses, we did not observe any significant differences in the cellular immune responses to vaccination. We used both ELISpot and flow cytometry to quantify SARS-CoV-2 spike protein subunits 1 and 2 specific cells, both showing very similar frequencies between study arms. This was not entirely unexpected, considering that differences in cellular responses have been reported before to be less pronounced than those in humoral responses [21]. The equivalent cellular immune responses observed in this study further support the notion that this reduced dosage likely induces potent immunity.

While acknowledging that this trial was not designed nor powered to study efficacy of protection, we did not observe an obvious difference in incidence of breakthrough infections between both study arms. It is important to consider, however, that these infections were most likely caused by the Delta variant of SARS-CoV-2, around six months after vaccination, while neutralizing titres against the Delta variant at 28 days post second dose were already very low for both the reduced and full dose arms. In other words, a possible difference in protection may have gone unnoticed due to a generally low incidence of infection during the first months following vaccination.

Based on waning antibody data and the emergence of new variants, high-income countries are providing third doses to the general population, and several countries have started administering fourth doses to specific groups with comorbidities. In the meantime, primary vaccination coverage in LIC remains very low. The COVAX program was founded in April 2020 with the specific aim to provide global equitable access to COVID-19 vaccines. The program has delivered approximately one billion vaccine doses to 144 participating countries, but current production capacity for COVID-19 vaccines does not cover the global needs, thus delaying the end of the pandemic. We acknowledge that the use of fractional doses is associated with a number of programmatic and operational challenges which may hamper its roll-out, such as the possible need for special syringes, adjustments of diluent volumes, or exacerbated vaccine hesitancy due to lower antibody levels [23,24]. Nevertheless, we believe that these challenges are rather limited and can still be solved relatively quickly, with the important exception of vaccine hesitancy which already presents a major barrier to effective roll-out even without fractional dosing. In addition, more structural strategies to speed up vaccination rates, such as strengthening logistics and building local vaccine production capacity, are important but will take a considerable amount of time to have an impact. Finally, the effects of fractional dosing on issues such as antibody waning and induction of variant cross-reactivity, amongst others, also need to be investigated further.

This study has several limitations, the first being the relatively limited sample size. A larger number of participants would have resulted in smaller confidence intervals around the GMRs, which might have impacted the conclusions on non-inferiority. Although males were underrepresented in this study, we do not believe this is a major limitation as immune responses to COVID-19 mRNA vaccination in healthy, younger subjects are only minimally gender-dependent and importantly, there is no basis to assume that fractional dosing would affect immune responses differently between males and females [25–27]. Secondly, the small proportion of previously infected participants in our study population (17/144, 12%) does not allow for a separate sensitivity analysis in this group. With record high COVID-19 incidences worldwide, the proportion of the population who experienced a past infection is rapidly growing, making analyses including previously infected people ever more relevant. In addition, breakthrough infections were not actively monitored by regular molecular testing. Therefore, we may have missed asymptomatic infections, which are not reported by the study participants. Thirdly, while protection from infection or disease has been convincingly correlated with titres of binding and neutralizing antibodies, as discussed previously, it is not possible to determine with certainty that the moderately lower titres observed in our study will translate to equally moderately lower efficacy of protection [15,28]. Fourth, while the interval of three weeks between both vaccine doses is recommended by the manufacturer, larger intervals are commonly adopted and have been shown to induce higher immune responses as compared to the shorter three week interval [29,30]. While we do not know whether such larger intervals would increase or decrease the difference between both dosing schedules, it is reasonable to assume that both dosages would benefit from an extended interval. As a result, with extended intervals, the lower dosed mRNA vaccine evaluated in this study might compare even better to the adenoviral vectored vaccine. Finally, a strength of the study in terms of global health relevance, is the relatively young age distribution of our cohort, ranging from 23 to 55 years old (median: 40). This makes our data especially relevant for the context where it can be applied most, i.e. LIC where age distribution is typically much lower than in high income countries (97% versus 81% of population below 65 years old, respectively) [31]. Nevertheless, further studies are needed including participants who are more reflective of the target population in terms of genetic background and environment.

To our knowledge, this is the only non-inferiority study reporting on fractional dosing of a COVID-19 mRNA vaccine so far. Our study shows moderately lower humoral responses and similar cellular immune responses after two reduced doses of 20μg compared to two full doses of 30μg of the BNT162b2 mRNA vaccine. Nevertheless, antibody responses to the reduced dose remain far superior to what has been published in the literature so far for marketed adenoviral vector vaccines with proven efficacy of protection against disease. Considering this and the important potential in accelerating vaccination rates in LIC, our findings support the need for larger non-inferiority trials on fractional dosing for primary vaccination as well as for follow-up booster vaccinations. The relevance of our findings goes beyond the current pandemic, as they highlight the potential of fractional dosing as a dose-sparing strategy in future epidemics and vaccine shortages.

## Supporting information

S1 Fig Decision tree to determine previous SARS-CoV-2 infection status at baseline. RBD = SARSCoV-2 receptor binding domain, ELISA = Enzyme linked immunosorbent assay, BAU = binding antibody units, MIA = multiplex immunoassay.
(TIF)

S2 Fig Flow cytometry cellular data in the per-protocol cohort at day 49 (28 days after second dose). SARS-CoV-2 spike protein subunit 1 and 2 (S1 and S2) specific T cell frequencies were measured in 42 infection naïve participants. A. Percentage of CD4+ and CD8+ T cells expressing CD154 (only in CD4), IFN-γ, IL-2, and TNF-α are depicted. Any: Percentage of cells positive for at least on the activation markers. A circle represents one test subject; the GMTs (95% CI) are shown in blue. LLOQ was fixed to 0.0001 and represented by a grey dashed line. B. Percentages are given for CD4+ and CD8+ T cells stimulated with S1 Wuhan or S2 Wuhan expressing at least one of the activation markers (corresponding to “any”). For continuous variables, p-values are reported using a linear mixed-effect model adjusted for gender, age and baseline SARS-CoV-2 anti-RBD IgG titre as fixed variables and location as random variable. Fisher’s exact test was used to report p-values for binary variables.
(TIF)

S3 Fig Adverse events. Reported local (A) and systemic (B) adverse events after the first (in blue) and second (in orange) vaccine dose, according to severity (mild/moderate/severe) and by study arm (20μg and 30μg) in the intention-to-treat cohort. Occurrence number (x-axis) and percentage (numbers inside bars) per AE calculated on the cohort are given.
(TIF)

S1 Table Immune responses by study arm at 28 days post second vaccine dose (Day 49) and non-inferiority analysis in the intention-to-treat cohort. (TIF)

S2 Table Humoral and cellular responses by study arm in the intention-to-treat cohort at the different time points. (PDF)

S3 Table Breakthrough infections. (TIF)

S4 Table Local and systemic adverse events in intention-to-treat and naïve only cohorts. (PDF)

S1 Appendix Study protocol. (PDF)

S2 Appendix Consort checklist. (PDF)

S3 Appendix Supplementary methods. (PDF)
