# Supplementary figures and images for: Safety and immunogenicity of a reduced dose of the BNT162b2 mRNA COVID-19 vaccine (REDU-VAC): A single blind, randomized, non-inferiority trial
Source: PLOS Glob Public Health. 2022 Dec 20;2(12):e0001308. doi: 10.1371/journal.pgph.0001308 (PMC10021431; doi:10.1371/journal.pgph.0001308)

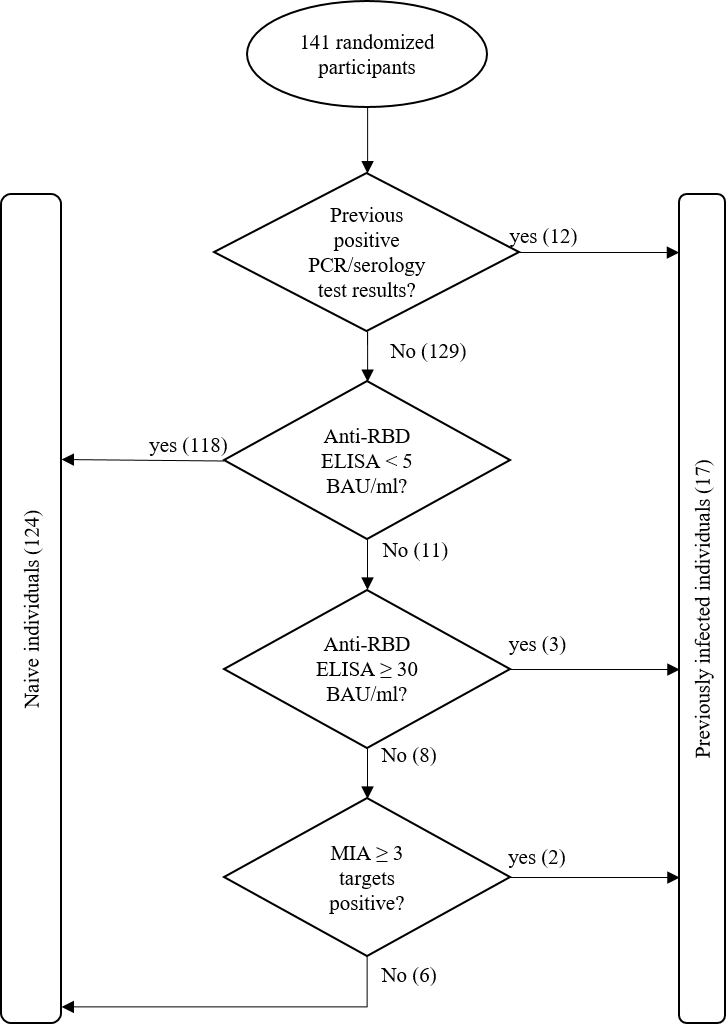

Supplement: S1 Fig — RBD = SARSCoV-2 receptor binding domain, ELISA = Enzyme linked immunosorbent assay, BAU = binding antibody units, MIA = multiplex immunoassay. (TIF) [file pgph.0001308.s001.tif]

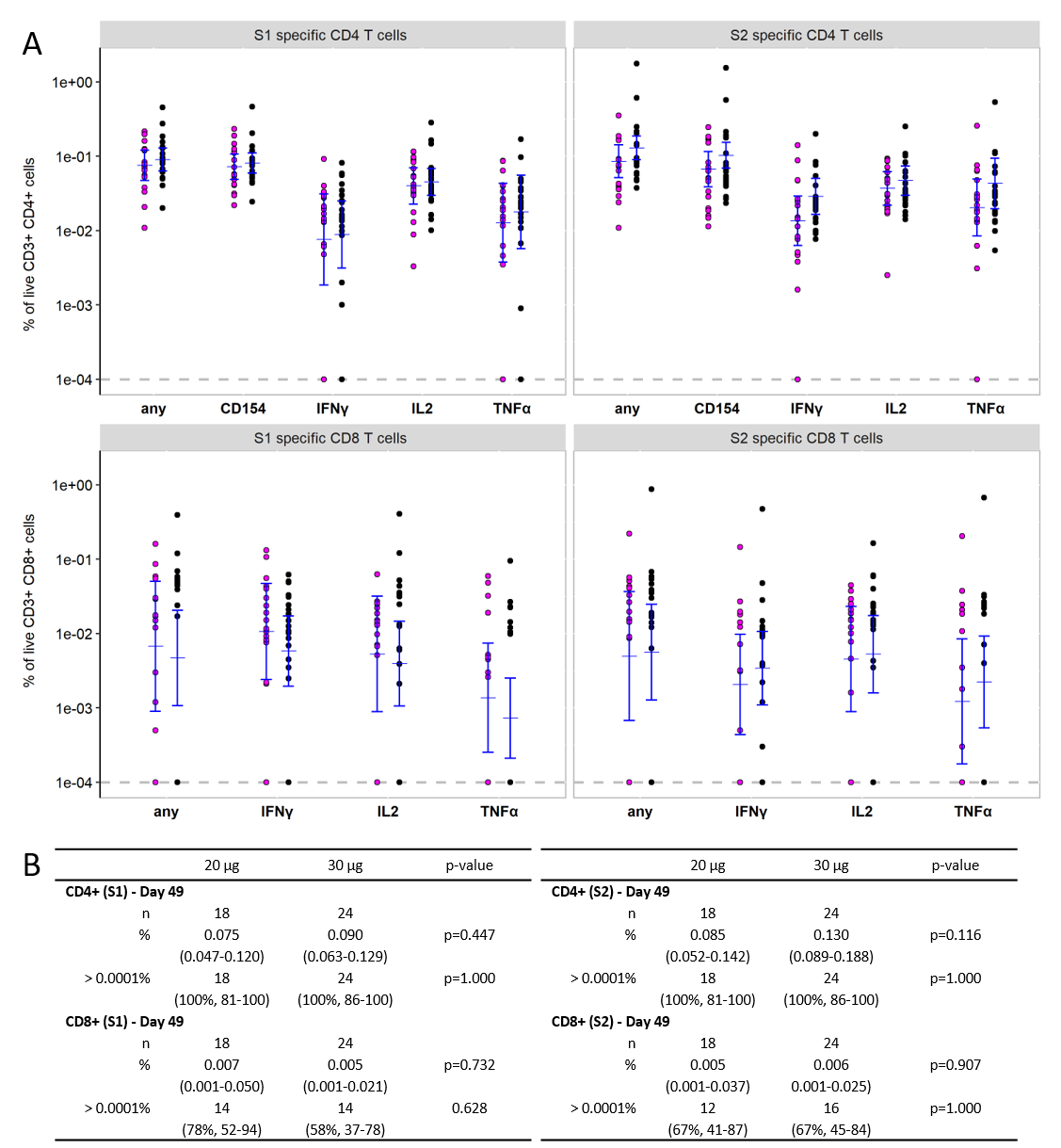

Supplement: S2 Fig — SARS-CoV-2 spike protein subunit 1 and 2 (S1 and S2) specific T cell frequencies were measured in 42 infection naïve participants. A. Percentage of CD4+ and CD8+ T cells expressing CD154 (only in CD4), IFN-γ, IL-2, and TNF-α are depicted. Any: Percentage of cells positive for at least on the activation markers. A circle represents one test subject; the GMTs (95% CI) are shown in blue. LLOQ was fixed to 0.0001 and represented by a grey dashed line. B. Percentages are given for CD4+ and CD8+ T cells stimulated with S1 Wuhan or S2 Wuhan expressing at least one of the activation markers (corresponding to “any”). For continuous variables, p-values are reported using a linear mixed-effect model adjusted for gender, age and baseline SARS-CoV-2 anti-RBD IgG titre as fixed variables and location as random variable. Fisher’s exact test was used to report p-values for binary variables. (TIF) [file pgph.0001308.s002.tif]

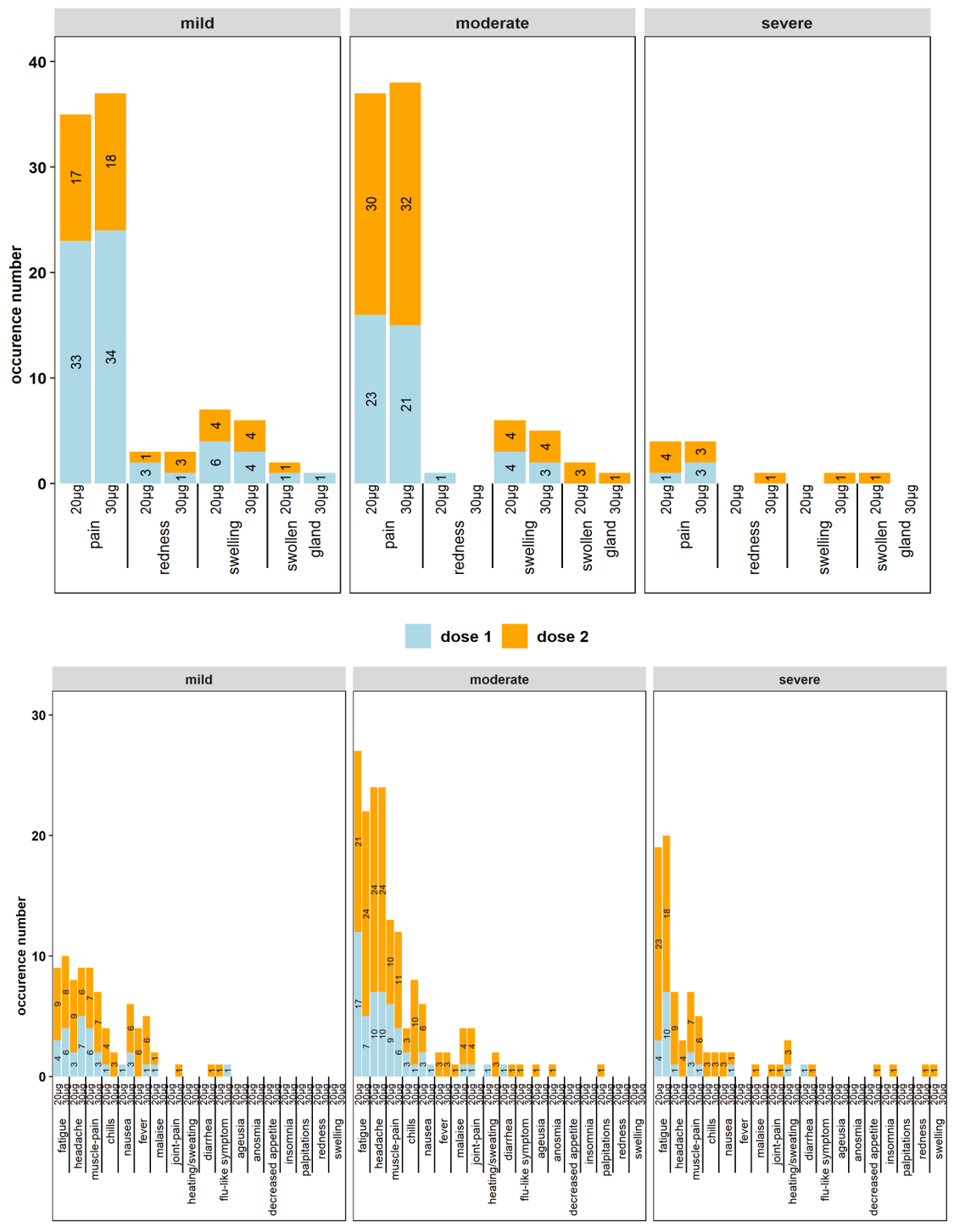

Supplement: S3 Fig — Reported local (A) and systemic (B) adverse events after the first (in blue) and second (in orange) vaccine dose, according to severity (mild/moderate/severe) and by study arm (20μg and 30μg) in the intention-to-treat cohort. Occurrence number (x-axis) and percentage (numbers inside bars) per AE calculated on the cohort are given. (TIF) [file pgph.0001308.s003.tif]

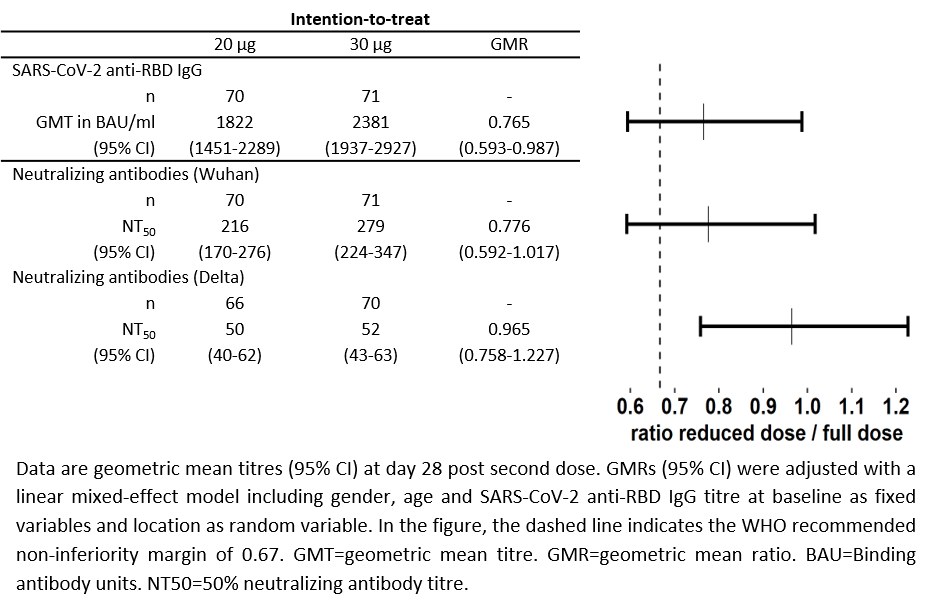

Supplement: S1 Table — (TIF) [file pgph.0001308.s004.tif]

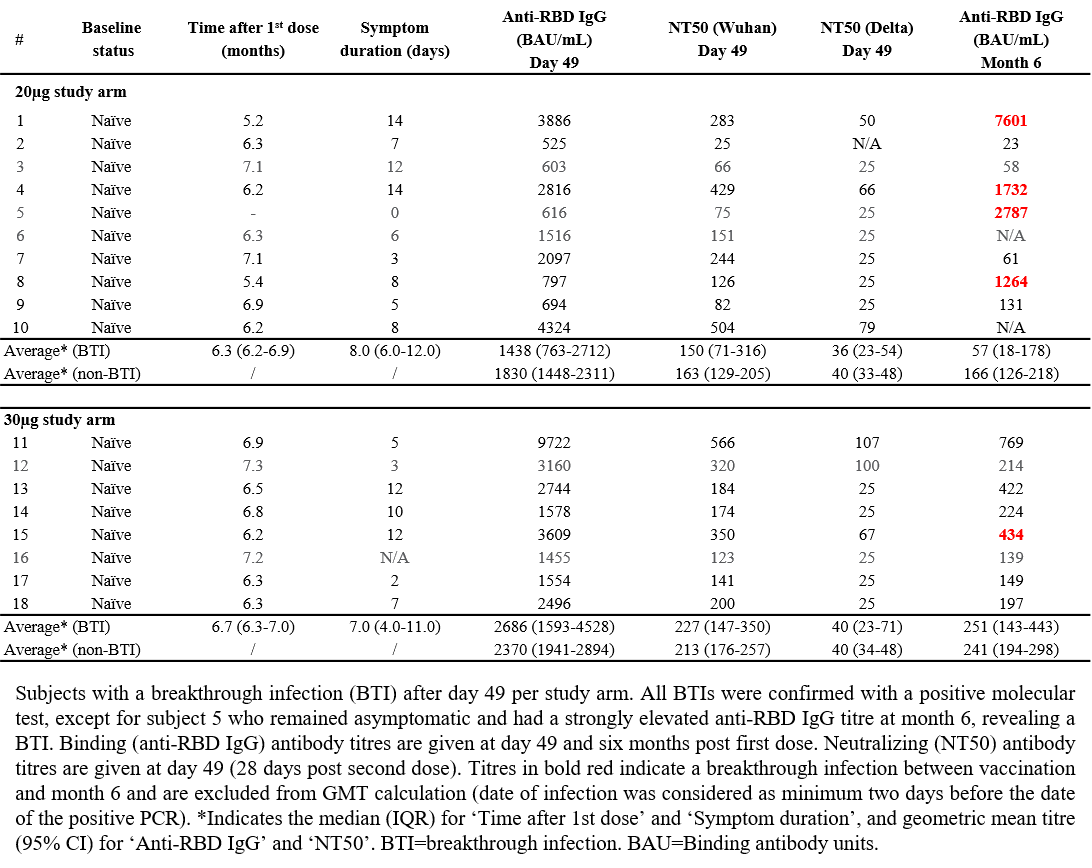

Supplement: S3 Table — (TIF) [file pgph.0001308.s006.tif]
