# Supplementary material for: Safety and immunogenicity of a reduced dose of the BNT162b2 mRNA COVID-19 vaccine (REDU-VAC): A single blind, randomized, non-inferiority trial
Source: PLOS Glob Public Health. 2022 Dec 20;2(12):e0001308. doi: 10.1371/journal.pgph.0001308 (PMC10021431; doi:10.1371/journal.pgph.0001308)
Supplement: S2 Table — (PDF) [file pgph.0001308.s005.pdf]

	Intention-to-treat		
	20 µg	30 µg	p-value
**SARS-CoV-2 anti-RBD IgG**			
**Day 0**			
n	70	71	
Concentration (BAU/ml)	10.9<br>(9.1-12.9)	9.9<br>(8.3-11.8)	p=0.310
> 5 BAU/ml	12<br>(17%, 9-28)	10<br>(14%, 7-24)	p=0.649
**Day 21**			
n	70	71	
Concentration (BAU/ml)	173<br>(125-240)	296<br>(218-400)	p=0.001
> 5 BAU/ml	69<br>(99% 92-100)	71<br>(100%, 95-100)	p=0.497
**Day 49**			
n	70	71	
Concentration (BAU/ml)	1822<br>(1451-2289)	2381<br>(1937-2927)	p=0.040
> 5 BAU/ml	70<br>(100%, 95-100)	71<br>(100%, 95-100)	p=1.000
**Month 6**			
n	59	67	
Concentration (BAU/ml)	190<br>(143-254)	272<br>(212-349)	p=0.019
> 5 BAU/ml	59<br>(100%, 94-100)	67<br>(100%, 95-100)	p=1.000
**Neutralizing antibodies**			
**Wuhan - Day 21**			
N	70	71	
NT_{50}	45<br>(36-56)	47<br>(39-58)	p=0.677
➢ 50	17<br>(24%, 15-36)	16<br>(23%, 13-34)	p=0.844
**Wuhan - Day 49**			
N	70	71	
NT_{50}	216<br>(170-276)	279<br>(224-347)	p=0.066
> 50	66<br>(94%, 86-98)	70<br>(99%, 92-100)	p=0.209
**Delta - Day 49**			
n	66	70	-
NT_{50}	50<br>(40-62)	52<br>(43-63)	p=0.767
> 50	31<br>(47%, 35-60)	31<br>(44%, 32-57)	p=0.863
**Omicron - Day 49**			
n	18	17	
NT_{50}	37<br>(28-48)	40<br>(30-54)	p=0.612
> 50	8<br>(44%, 22-69)	5<br>(29%, 10-56)	p=0.489

	Intention-to-treat		
	20 µg	30 µg	p-value
**SARS-CoV-2 anti-RBD IgG**			
**Day 0**			
n	70	71	
Concentration (BAU/ml)	10.9<br>(9.1-12.9)	9.9<br>(8.3-11.8)	p=0.310
> 5 BAU/ml	12<br>(17%, 9-28)	10<br>(14%, 7-24)	p=0.649
**Day 21**			
n	70	71	
Concentration (BAU/ml)	173<br>(125-240)	296<br>(218-400)	p=0.001
> 5 BAU/ml	69<br>(99% 92-100)	71<br>(100%, 95-100)	p=0.497
**Day 49**			
n	70	71	
Concentration (BAU/ml)	1822<br>(1451-2289)	2381<br>(1937-2927)	p=0.040
> 5 BAU/ml	70<br>(100%, 95-100)	71<br>(100%, 95-100)	p=1.000
**Month 6**			
n	59	67	
Concentration (BAU/ml)	190<br>(143-254)	272<br>(212-349)	p=0.019
> 5 BAU/ml	59<br>(100%, 94-100)	67<br>(100%, 95-100)	p=1.000
**Neutralizing antibodies**			
**Wuhan - Day 21**			
N	70	71	
NT_{50}	45<br>(36-56)	47<br>(39-58)	p=0.677
➢ 50	17<br>(24%, 15-36)	16<br>(23%, 13-34)	p=0.844
**Wuhan - Day 49**			
N	70	71	
NT_{50}	216<br>(170-276)	279<br>(224-347)	p=0.066
> 50	66<br>(94%, 86-98)	70<br>(99%, 92-100)	p=0.209
**Delta - Day 49**			
n	66	70	-
NT_{50}	50<br>(40-62)	52<br>(43-63)	p=0.767
> 50	31<br>(47%, 35-60)	31<br>(44%, 32-57)	p=0.863
**Omicron - Day 49**			
n	18	17	
NT_{50}	37<br>(28-48)	40<br>(30-54)	p=0.612
> 50	8<br>(44%, 22-69)	5<br>(29%, 10-56)	p=0.489

Data are geometric means (95% CI) for continuous variables, and n (%, 95% CI) for binary values. The LLOQ for the SARS-CoV-2 anti-RBD IgG titre was 5 BAU/mL, NT50=50 for the live virus neutralization assay, 54 and 66 cells/million PBMCs for S1 and S2, respectively, for ELIspot, and 0.0001% for flow cytometry. The mean in IFN-γ ELISpot was obtained from three replicate values. For continuous variables, p-values are reported using a linear mixed-effect model adjusted for gender, age and baseline infection status (for day 0 data) or baseline SARS-CoV-2 anti-RBD IgG titre (for day 21/49 and month 6 data) as fixed variables and location as random variable. Fisher's exact test was used to report p-values for binary variables. BAU=Binding antibody units. NT50=50% neutralizing antibody titre.
