# Supplementary material for: Safety and immunogenicity of a reduced dose of the BNT162b2 mRNA COVID-19 vaccine (REDU-VAC): A single blind, randomized, non-inferiority trial
Source: PLOS Glob Public Health. 2022 Dec 20;2(12):e0001308. doi: 10.1371/journal.pgph.0001308 (PMC10021431; doi:10.1371/journal.pgph.0001308)
Supplement: S4 Table — (PDF) [file pgph.0001308.s007.pdf]

	Intention-to-treat			Naive		
	**20 µg**	**30 µg**	**p-value**	**20 µg**	**30 µg**	**p-value**
Participants, n	70	71		60	64	
Total number of local adverse events						
mild	47	47	0.95*	41	42	0.97*
moderate	46	44		39	41	
severe	5	6		5	4	
Total number of systemic adverse events						
mild	38	42	0.63*	29	39	0.23*
moderate	87	76		74	68	
severe	44	37		43	33	
**Local adverse events, dose 1**						
none	27 (39%)	29 (41%)	0.86	23 (38%)	26 (41%)	0.86
pain						
mild	23 (33%)	24 (34%)	1*	19 (32%)	22 (34%)	0.90*
moderate	16 (23%)	15 (21%)		14 (23%)	14 (22%)	
severe	1 (1%)	2 (3%)		1 (2%)	1 (2%)	
total	40 (57%)	41 (59%)	1	34 (57%)	37 (58%)	1
redness						
mild	2 (3%)	1 (1%)	1*	2 (3%)	1 (2%)	1*
moderate	1 (1%)	0		1 (2%)	0	
total	3 (4%)	1 (1%)	0.37	3 (5%)	1 (2%)	0.35
swelling						
mild	4 (6%)	3 (4%)	1*	3 (5%)	2 (3%)	1*
moderate	3 (4%)	2 (3%)		2 (3%)	2 (3%)	
total	7 (10%)	5 (7%)	0.56	5 (8%)	4 (6%)	0.74
swollen glands						
mild	1 (1%)	1 (1%)	1	1 (2%)	1 (2%)	1
**Local adverse events, dose 2**						
none	30 (42%)	29 (41%)	0.87	24 (40%)	26 (41%)	1
pain						
mild	12 (17%)	13 (18%)	0.94*	12 (20%)	12 (19%)	0.53*
moderate	21 (30%)	23 (32%)		17 (28%)	21 (33%)	
severe	3 (4%)	2 (3%)		3 (5%)	1 (2%)	
total	36 (51%)	38 (54%)	0.87	32 (53%)	34 (53%)	1
redness						
mild	1 (1%)	2 (3%)	1*	1 (2%)	2 (3%)	1*
severe	0	1 (1%)		0	1 (2%)	
total	1 (1%)	3 (4%)	0.62	1 (2%)	3 (5%)	0.62
swelling						
mild	3 (4%)	3 (4%)	1*	2 (3%)	2 (3%)	1*
moderate	3 (4%)	3 (4%)		3 (5%)	3 (5%)	
severe	0	1 (1%)		0	1 (2%)	
total	6 (9%)	7 (10%)	1	5 (8%)	6 (9%)	1
swollen glands						
mild	1 (1%)	0	1*	1 (2%)	0	1*
moderate	2 (3%)	1 (1%)		2 (3%)	1 (2%)	
severe	1 (1%)	0		1 (2%)	0	
total	4 (6%)	1 (1%)	0.21	4 (7%)	1 (2%)	0.20

		Intention-to-treat			Naive	
	**20 µg**	**30 µg**	**p-value**	**20 µg**	**30 µg**	**p-value**
Participants, n	70	71		60	64	
**Systemic adverse events, dose 1**						
none	39 (56%)	43 (61%)	0.61	34 (57%)	40 (63%)	0.58
chills						
mild	1 (1%)	0	1*	1 (2%)	0	1*
moderate	2 (3%)	1 (1%)		2 (3%)	0	
total	3 (4%)	1 (1%)	0.37	3 (5%)	0	0.11
diarrhea						
moderate	1 (1%)	0	1*	0	0	1*
severe	1 (1%)	0		1 (2%)	0	
total	2 (3%)	0	0.24	1 (2%)	0	0.48
fatigue						
mild	3 (4%)	4 (6%)	0.11*	1 (2%)	4 (6%)	0.16*
moderate	12 (17%)	5 (7%)		10 (17%)	5 (8%)	
severe	3 (4%)	7 (10%)		3 (5%)	5 (8%)	
total	18 (26%)	16 (23%)	0.70	14 (23%)	14 (22%)	1
fever						
mild	0	1 (1%)	1	0	0	1
flu-like symptom						
mild	0	1 (1%)	1	0	1 (2%)	1
headache						
mild	2 (3%)	5 (7%)	0.49*	2 (3%)	4 (6%)	0.62*
moderate	7 (10%)	7 (10%)		6 (10%)	5 (8%)	
severe	1 (1%)	0		1 (2%)	0	
total	10 (14%)	12 (17%)	0.82	9 (15%)	9 (14%)	1
heating/sweating						
moderate	1 (1%)	0	1*	1 (2%)	0	1*
severe	1 (1%)	0		1 (2%)	0	
total	2 (3%)	0	0.24	2 (3%)	0	0.23
joint pain						
moderate	1 (1%)	0	0.50	1 (2%)	0	0.48
malaise						
mild	1 (1%)	0	1*	1 (2%)	0	1*
moderate	0	1 (1%)		0	1 (2%)	
total	1 (1%)	1 (1%)	1	1 (2%)	1 (2%)	1
muscle pain						
mild	4 (6%)	2 (3%)	1*	3 (5%)	2 (3%)	1*
moderate	6 (9%)	4 (6%)		4 (7%)	2 (3%)	
severe	2 (3%)	1 (1%)		2 (3%)	0	
total	12 (17%)	7 (10%)	0.23	9 (15%)	4 (6%)	0.15
nausea						
mild	1 (1%)	2 (3%)	1*	1 (2%)	1 (2%)	1*
moderate	2 (3%)	1 (1%)		2 (3%)	1 (2%)	
severe	0	1 (1%)		0	1 (2%)	
total	3 (4%)	4 (6%)	1	3 (5%)	3 (5%)	1

		Intention-to-treat			Naive	
	20 µg	30 µg	p-value	20 µg	30 µg	p-value
Participants, n	70	71		60	64	
**Systemic adverse events, dose 2**						
none	21 (30%)	27 (38%)	0.38	17 (28%)	23 (36%)	0.44
ageusia						
moderate	1 (1%)	0	0.50	1 (2%)	0	0.48
anosmia						
moderate	1 (1%)	0	0.50	1 (2%)	0	0.48
chills						
mild	3 (4%)	2 (3%)	0.38*	2 (3%)	2 (3%)	0.51*
moderate	2 (3%)	7 (10%)		2 (3%)	7 (11%)	
severe	2 (3%)	2 (3%)		2 (3%)	2 (3%)	
total	7 (10%)	11 (15%)	0.45	6 (10%)	11 (17%)	0.30
decreased appetite						
severe	0	1 (1%)	0.50	0	1 (2%)	1
diarrhea						
mild	0	1 (1%)	1*	0	1 (2%)	1*
moderate	0	1 (1%)		0	1 (2%)	
severe	0	1 (1%)		0	1 (2%)	
total	0	3 (4%)	0.24	0	3 (5%)	0.24
fatigue						
mild	6 (9%)	6 (8%)	0.86*	5 (8%)	6 (9%)	0.65*
moderate	15 (21%)	17 (24%)		13 (22%)	16 (25%)	
severe	16 (23%)	13 (18%)		16 (27%)	12 (19%)	
total	37 (53%)	36 (51%)	0.87	34 (57%)	34 (53%)	0.72
fever						
mild	4 (6%)	4 (6%)	1*	3 (5%)	4 (6%)	1*
moderate	2 (3%)	2 (3%)		2 (3%)	2 (3%)	
total	6 (9%)	6 (8%)	1	5 (8%)	6 (9%)	1
flu-like symptom						
mild	1 (1%)	0	1*	1 (2%)	0	1*
moderate	1 (1%)	0		0	0	
total	2 (3%)	0	0.24	1 (2%)	0	0.48
headache						
mild	6 (9%)	4 (6%)	0.67*	6 (10%)	4 (6%)	0.61*
moderate	17 (24%)	17 (24%)		14 (23%)	16 (25%)	
severe	6 (9%)	3 (4%)		6 (10%)	3 (5%)	
total	29 (41%)	24 (34%)	0.39	26 (43%)	23 (36%)	0.46
heating/sweating						
moderate	0	2 (3%)	0.33*	0	2 (3%)	0.33*
severe	2 (3%)	0		2 (3%)	0	
total	2 (3%)	2 (3%)	1	2 (3%)	2 (3%)	1
insomnia						
severe	0	1 (1%)	0.50	0	1 (2%)	1
joint pain						
mild	0	1 (1%)	0.20*	0	1 (2%)	0.20*
moderate	3 (4%)	0		3 (5%)	0	
severe	1 (1%)	1 (1%)		1 (2%)	1 (2%)	
total	4 (6%)	2 (3%)	0.44	4 (7%)	2 (3%)	0.43

		Intention-to-treat			Naive	
	20 µg	30 µg	p-value	20 µg	30 µg	p-value
Participants, n	70	71		60	64	
**General adverse events, dose 2 (continued)**						
malaise						
mild	1 (1%)	0	0.40*	0	0	1*
moderate	1 (1%)	3 (4%)		1 (2%)	3 (5%)	
severe	1 (1%)	0		0	0	
total	3 (4%)	3 (4%)	1	1 (2%)	3 (5%)	0.62
muscle pain						
mild	5 (7%)	5 (7%)	1*	3 (5%)	5 (8%)	0.81*
moderate	7 (10%)	8 (11%)		7 (12%)	7 (11%)	
severe	5 (7%)	4 (6%)		5 (8%)	4 (6%)	
total	17 (24%)	17 (24%)	1	15 (25%)	16 (25%)	1
nausea						
mild	0	4 (6%)	0.01*	0	4 (6%)	0.05*
moderate	4 (6%)	0		3 (5%)	0	
severe	2 (3%)	1 (1%)		2 (3%)	1 (2%)	
total	6 (9%)	5 (7%)	0.76	5 (8%)	5 (8%)	1
palpitations						
moderate	1 (1%)	0	0.50	1 (2%)	0	0.48
redness						
severe	0	1 (1%)	0.50	0	1 (2%)	1
swelling						
severe	1 (1%)	0	0.50	1 (2%)	0	0.48

Fisher's exact test was used to report p-values. *test between the levels of the scale of the present symptoms (mild/moderate/severe).
