# Supplementary material for: Safety and immunogenicity of a reduced dose of the BNT162b2 mRNA COVID-19 vaccine (REDU-VAC): A single blind, randomized, non-inferiority trial
Source: PLOS Glob Public Health. 2022 Dec 20;2(12):e0001308. doi: 10.1371/journal.pgph.0001308 (PMC10021431; doi:10.1371/journal.pgph.0001308)
Supplement: S1 Appendix — (PDF) [file pgph.0001308.s008.pdf]

Safety and Immunogenicity of a Reduced Dose of the BioNTech/Pfizer BNT162b2 Vaccine in a Healthy Population (REDU-VAC)

A randomized multicenter interventional clinical COVID-19 vaccination trial

1. General information

This phase IV dose-optimization study will be coordinated by the service Epidemiology of Infectious Diseases and Cancer center (contact: Mieke Goossens, Pieter Pannus) of the scientific directorate of Epidemiology and public health, Sciensano. Laboratory analyses will be performed by the service Immune response (contact: Isabelle Desombere) and Viral diseases (contact: Cyril Barbezange, Isabelle Thomas) of the scientific directorate of Infectious diseases in humans of Sciensano. The study will be executed in collaboration with Mensura EDPB (contact: Marie-Noëlle Schmickler and Mathieu Verbrugghe), the Virology Unit of the Institute of Tropical Medicine Antwerp (contact: Kevin Ariën), the Institute for Medical Immunology of ULB, Campus Erasme, (contact: Arnaud Marchant) and Campus Gosselies (contact: Stanislas Goriely).

2. Background

COVID-19 vaccines are being rolled out in many countries all over the world. Promising efficacy data from phase three vaccination trials are being confirmed with real-world data from countries like Israel and the United Kingdom where large proportions of the population have already been vaccinated (1–3).

Vaccine supply has proven an important limiting factor for the speed of vaccination campaigns. Indeed, vaccine demand largely surpasses production capacity of the different manufacturers. In order to cope with this scarcity, different strategies have been proposed and implemented, including a delayed second dose and a single instead of dual dose for previously infected people. Another possible strategy is a reduction of the vaccine dose for those population groups who generally have better immunologic vaccine responses, which is the subject of this clinical trial (4). Indeed, in this study we will investigate immune responses to a reduced dose of the BNT162b2 mRNA vaccine of BioNTech/Pfizer.

Data from a dose-escalating phase 1 trial in healthy adults 18 to 85 years of age comparing two doses of 10µg, 20µg, 30µg and 100µg indicated that a dose of 30µg of BNT162b2 achieved the best immune response in participants of all ages (5). As a result, a phase2/3 trial was conducted and the vaccine was finally marketed at a dosage of 30µg (6). These immune responses were age dependent, however. While people aged 65-85 years (N=24) had markedly better responses with 30µg as compared to 20µg, this was not the case for people aged 18-55 years (N=24). Indeed, SARS-CoV-2 specific binding and neutralizing antibody titers were even slightly higher in the 20µg group as compared to the 30µg group. We therefore propose to conduct a clinical trial investigating immune responses comparing a 20µg versus 30µg dose of BNT162b2 in a larger cohort of 150 subjects..

3. Objectives and outcomes

We aim to include 150 adults aged 18-55 years from five Mensura EDPB sites. These will be equally randomized in two study arms receiving two doses of:

- Arm 1: 20µg BNT162b2
- Arm 2: 30µg BNT162b2

We aim to include up to 25 previously SARS-CoV-2 infected subjects per arm. The objectives of this study include assessing the immunogenicity (both humoral and cellular), safety and reactogenicity of a reduced vaccine dose.

3.1. PRIMARY OBJECTIVE AND OUTCOME

- The primary objective is to prove non-inferiority of immunogenicity of a reduced BNT162b2 vaccine dose (20µg) versus the reference vaccine dose (30µg).

- The primary outcome is the geometric mean titer (GMT) of binding (IgG) antibodies specific to the receptor binding domain (RBD) of SARS-CoV-2 at four weeks after the second dose.

Data from the literature and from our own PICOV-VAC data indicate strong correlation between titers of RBD binding antibodies and neutralizing antibodies. As methods to measure RBD binding antibodies can be implemented more rapidly than neutralizing antibody assays, we consider that RBD binding antibodies are a good surrogate for neutralizing antibodies and can therefore be used as a primary endpoint for this study.

3.2. SECONDARY OBJECTIVES AND OUTCOMES

- GMT of RBD-specific binding antibodies at the time of the second dose, six months and one year after the first dose.
- GMT of neutralizing antibody titers against wild type and variant SARS-CoV-2 viruses at four weeks after second dose administration.
- Cellular immunity parameters (i.e. Memory B-cell responses, T-cell responses, etc) at all clinically and biologically relevant time points.
- Safety and reactogenicity of the different vaccine regimens as defined by the severity, duration and amount of adverse events experienced after each vaccine dose.

4. Methods

This is a randomized interventional clinical trial in healthy subjects organized at five sites of Mensura EDPB.

Inclusion criteria

- Employed by Mensura EDBP (should be an employee at least until the end of the study)
- Aged 18-55 years

Exclusion criteria

- Previously vaccinated against COVID-19
- Pregnant/breastfeeding women

Discontinuation criteria

All participants retain the right to end their participation in the study at any point in time. Participants who fail to provide the necessary information through the questionnaires, will be discontinued from the study, since any correlation analysis with biological measurements becomes impossible.

The primary objective of the study is to prove non-inferiority of anti-RBD binding antibody titers four weeks after the second dose of the 20µg versus 30µg study arm. If the primary outcome of the study reveals an inferiority of the 20µg dose, participants in the 20µg dose arm will be offered a third, 30µg dose of the study vaccine.

Although no correlate of protective immunity has been validated yet, a relationship between vaccine-induced SARS-CoV-2 antibodies and protection has been proposed (7).

Sample size

The primary analysis is a non-inferiority comparison at 28 days after the second dose, for the GMT of antibodies binding to the RBD of SARS-COV-2, comparing the reference dose (30µg BNT162b2) with the reduced dose (20µg BNT162b2).

Currently available data from published and ongoing COVID-19 vaccination trials indicate that:

- RBD-specific antibody titers (GMT, arbitrary units) 28 days after the second dose of BNT162b2 equals 2125. The standard deviation on the log scale (base 10) is 0.27 (PICOV-VAC trial).

The following assumptions are made in the sample size calculations:

- The non-inferiority margin is -0.15 absolute difference of GMT on log scale (base 10), between a reduced dose and regular dose vaccine schedule.
- The standard deviation of GMT on the log10 scale is 0.27 for BNT162b2, based on the currently available data.
- The true difference of GMT on log10 scale is 0.
- A two-sided 2.5% family-wise error rate (at cohort level).

Based on these assumptions, the comparison between both doses will need a minimum of 50 participants (infection naïve) per group to achieve 90% power. In order to be able to better evaluate reactogenicity however, we aim for a larger sample size.

Trial randomization

Subject randomization will be done using statistical software (RStudio). The system will randomly allocate the 150 participants across both study arms (20µg and 30µg) irrespective of their previous SARS-CoV-2 infection status. The recruitment and randomization process will aim to have a balanced representation of age and gender across the study arms. Therefore stratified randomization will be used based on age and gender.

In addition, 20 participants will be randomly selected per study arm (40 in total) from which extra (heparinized) blood will be collected for in-depth cellular immunogenicity analyses (see table 1). This random selection will be done using the same statistical software (RStudio).

Blinding/unblinding

Everyone involved in the study will be blinded to the study arm allocation of each participant, except for the study nurses administering the vaccine.

When the primary outcome of the study has been reached (28 days after the second dose), an interim analysis will be conducted to determine whether the reduced vaccine dose induces an inferior immune response as compared to the regular dose or not. In case of non-inferiority, there will be no need for unblinding before the end of the trial. In case of inferiority, study participants will be unblinded, informed about which study arm they were allocated to and offered a third dose (of 30µg) of BNT162b2.

Study flow

Participant information and consent procedure

A research nurse checks the study eligibility criteria and informs the candidates on the possibility to participate in the study. Participant information and consent includes explaining that the national number will be recorded on site by the investigator for possible later data linkage. The participant information and consent will also include that a trusted third party (TTP) will receive and use the national number to link with administrative data. This data linkage is planned to obtain a more complete data set that will be used for the analysis of possible hospitalization and other medical activities billed to the health insurance.

Participant information and consent includes explaining that the email and mobile number from study participants will be recorded in a separate and protected form in the database hosted by the

occupational physician. These data are only used to send emails and text messages to the participant for this study.

After having had the possibility to think and discuss about their participation, the participant gives written informed consent and provides the research nurse with their email address and telephone number that they intend to use the next 12 months.

Baseline activities

After the participant has given written informed consent, the research nurse completes the Subject ID log with participant's name, national number and study number. The participant national number is collected and kept on site by the research nurse in the Subject ID log. Each of the participating sites will transfer this local Subject ID log at study closure to the TTP for data linkage, following the procedure detailed in the authorization of the Information Security Committee.

The research nurse creates a new participant in the study eCRF (hosted on LimeSurvey) and enters the date of consent, gender, year of birth, height, weight. They will also register whether the participant has had a past SARS-CoV-2 infection (i.e. positive nasopharyngeal swab, positive serology test).

Once the information is entered by the research nurse in the eCRF, the participant automatically receives an email (subject "Clinical Trial Message" and starting with "Dear Madam, Dear Sir") with a link to the data entry screens for the patient-reported-outcomes (PROMs). In order to have a second identity check, the participant first enters year of birth and their study number.

The research nurse can view the PROMs of his/her subjects and only exceptionally make corrections upon explicit request by the participant. Each such change will be logged and needs a justification. At all times the research nurse can indicate in the system the early end of study (and stop sending emails to the participant to complete the PROMs), e.g. in case of withdrawal of consent, if known to the research nurse. In case of withdrawal of consent, data already collected will be kept in the study data.

Vaccination

Depending on the randomization, participants will either receive two doses of 20µg or two doses of 30µg of the BioNTech/Pfizer SARS-CoV-2 mRNA vaccine, with a three week interval between both doses.

Sampling visits

An overview of the different sample types and volumes at each study visit is summarized in table 1. Venous blood will be collected on the day of the first and second dose as well as four weeks after the second dose, and six months and one year after the first dose. Five mL of blood will be collected from all study participants at all study visits. From a random selection of 20 participants per study arm (40 in total), 36mL of heparinized blood will be collected on the day of the first dose as well as four weeks after the second dose and six months after the first dose.

The sampling activity (date, sample types collected) is documented in the eCRF. Samples are handled as detailed in the sampling manual.

One week after each vaccination dose, participants will be invited by email and text message by the research nurse to fill in a questionnaire collecting information on the reactogenicity of the vaccine.

Monitoring of adverse reactions

Suspected unexpected serious adverse reactions, serious adverse reactions, and adverse reactions with grade equal or more than 3 will be monitored for the duration of the study period. The study nurse will complete the reporting form (annex 4) and forward this to the study coordinator, who in turn will report this immediately to the Belgian Federal Agency for Medicines and Health Products.

End of the trial

The trial will end when the last study participant has been sampled one year after first dose administration.

4.1. DATA MANAGMENT

Safety

Based on previously published phase 2 and 3 data of both vaccines that will be used in this study, very few to no serious adverse events are expected (6,8). Besides vaccination, no safety issues linked with the other study procedures are expected either. As a consequence this is considered a low risk study.

Data collection

Laboratory data (samples) and epidemiological data (questionnaire) will be collected as described above. Each participant will receive a unique identifier code at the start of the study that will be used both on the samples and the questionnaires through the whole study period.

Laboratory data

Blood samples will be collected as indicated in table 1. Serum will be isolated from serum gel blood collection tubes and will be used for:

- SARS-CoV-2 specific binding antibody quantification: using an *in-house* anti-RBD IgG ELISA.
- Neutralizing antibody capacity measurement against the wild type Wuhan strain and at least one variant of concern: using a virus neutralization assay.

Peripheral Blood Mononuclear Cells (PBMC) will be isolated from heparinized blood on the day of blood draw and will be stored in liquid nitrogen for further analyses. These include:

- Memory B-cell responses: using B-cell ELISpot of flow cytometry
- T-cell responses: using intra-cellular cytokine staining

Samples will be collected at the research sites by a research nurse of Mensura EDPB.

Epidemiological data

Socio-demographic characteristics and SARS-CoV-2 initial status (date of last PCR test) will be collected by the research nurse at the time of inclusion and will be pseudonymized (without the identity of the participant but with a unique participant code, see further) transferred to Sciensano after informed consent of the participant was obtained. Additional questionnaires will be filled in by the participants given time points (see Table 1), and will provide information on reactogenicity and severity of the adverse events. All questionnaires will be completed through a secured online application (see further).

Data flow and management

Blood collection tubes as well as labelling stickers will be provided by Sciensano. For the entirety of the study, material will be provided in batch and a transport will be organized between Sciensano and the research sites. After collection, samples will be handled following the appropriate SOP.

Epidemiological and laboratory data will be linked via a unique code assigned to each participant. This code will start with the first letters of the research site location followed by a three digit number (e.g. “BRU_001” for the first participant, “BRU_002” for the second one, and so forth).

A sticker labelled with this code will be affixed by the research nurse on each tube at the time of sampling, and the same corresponding code will be entered in each questionnaire, enabling the link for data analysis. For the planned days, the labels will also include an identifier of this point (e.g. “D00”, “D28” etc). This unique code must stay the same during all the duration of follow-up and a list of all participants and their assigned codes will be kept in a secure and protected way by the occupational physician.

Questionnaires are filled in online through LimeSurvey. Before sampling takes place, an email will be sent to the participant containing a questionnaire link and the unique participant code. It will take around

five minutes for the participant to fill in the questionnaire. If the questionnaire is not filled in yet at the moment of sampling, it will be filled in together with the research nurse. A confirmation email will be sent to the research nurse when a questionnaire is correctly received. LimeSurvey is a web application running on a server located within Sciensano's datacenter. The application and its associated database are located on the same server. It means there are no data located outside Sciensano. Access to the administration of the LimeSurvey application is restricted to a limited number of people involved in the administrative management of the survey, who are authenticated with a username and password. All files will be kept on the Sciensano SQL server with restricted access.

Login to the shared server is password controlled. Each user/investigator or research nurse will receive a personal login name and password and will have a specific role which has predefined restrictions on what is allowed on the server. Furthermore, users will only be able to see data of subjects of their own site. Any activity in the software is traced and transparent via log files.

Direct access to all study records, including source documents and the eCRF will be granted to authorized representatives from the Sponsor, host institution and the regulatory authorities to permit study-related monitoring, audits and inspections. The Data Manager will review the eCRFs to make certain that all items have been completed. Incorrect or inappropriate entries in the eCRFs will be returned to the research nurse for correction (data queries), if not already captured using automated data entry checks. Subject privacy must be respected at all times, in accordance to GDPR, GCP and all other applicable local regulations. The investigator/study team should immediately notify the sponsor if he or she has been contacted by a regulatory agency concerning an upcoming inspection.

The data capture software analysis, development and testing will be performed according to the procedures of the Sponsor. Quality check of data management and data entry will be performed in accordance with the standard operating procedures of the Sponsor.

Analysis

Data analysis

Analysis of all data collected within this study will be performed by Sciensano. Questionnaire responses will always be coded. All analyses will be performed in R (R Core Team (2018), available from https://www.R-project.org/), STATA or SAS.

Meaningful laboratory results of each participant will be communicated to the research nurse who will be able to communicate them to the participants. This includes the anti-SARS-CoV-2 antibody status at the primary endpoint and at the end of the study.

As already mentioned, these tests are for research and not diagnostic purposes, and depending on lab capacity, there will be a delay in the communication of the results. The global results of the study will be communicated to each participating center at the end of the study in the form of a report and/or presentations.

Statistical analyses

The analysis described here will be coordinated by the Sponsor. Any later analyses on the linked data will also be conducted by the Sponsor.

All of the participants who meet the eligibility criteria will be included in the main analysis. Descriptive analyses will be used to report participant characteristics. The level of antibody titers in each of the study arms will be analyzed with the Kruskal-Wallis test, while participant demographics (e.g. age, gender) will be taken into account (confounders). Furthermore, risk factors and correlations with antibody detectability will be evaluated. P-values below 0.05 will be considered statistically significant.

5. Ethics and Privacy Protection

5.1. INDEPENDENT ETHICS COMMITTEE AND INFORMED CONSENT

Prior to study start, this protocol will be reviewed and approved by an Independent Ethics Committee (IEC). Written, dated and signed informed consent has to be obtained from each eligible subject prior to inclusion in the study. A sample Participant Information/Informed Consent Form has been prepared (see appendix).

5.2. DATA LINKAGE

An electronic case report form (eCRF) will be used for data collection. Subject confidentiality will be maintained at all times, within the legal constraints. The exported participant reported outcome measures (PROMs) dataset will contain pseudonymized participant data. A separate instrument (“form”) with separate restricted access will contain direct identifiable subject data (subject’s email address and mobile phone number of the participant) such that emails and additional reminders by text message can be sent to the subject. Separately from the study database, the local investigator will store on their Mensura EDPB facility a Subject ID log (e.g. in a spreadsheet with restricted access, GDPR compliant) containing the national number of the participant and the unique participant number for a possible later data linkage by a trusted third party (TTP), f.e. eHealth. The linkage will follow a procedure approved by the competent chamber of the Information Security Committee.

After the Ethics Committee has approved this protocol, the Sponsor will introduce a request to the competent chamber of the Information Security Committee for the linkage of the identifiable study data including participant reported outcome measures (PROMs), sickness fund (IMA/AIM) financial data (RIZIV/INAMI expenses). Only after the approval of this request, the parties will start data linkage. The principle investigator will guarantee the data protection.

6. Organization of the research project

6.1. TIMELINE

The anticipated start date of the study is May 3rd 2021. The end of the trial is anticipated to be the beginning of June 2022.

6.2. STUDY PARTIES AND RESPONSIBILITIES

Study sponsor: Sciensano

Study contact and principle investigator is Dr. Maria Goossens, Sciensano.

The sponsor has in-house expertise in the lab tests.

The sponsor has in-house expertise with LimeSurvey for both the eCRFs and the collection of PROMs. Data will be captured on a secured server.

The biostatistician of the Sponsor will perform the study data analysis.

The independent Ethic committee: ULB Erasme

The current epidemic context justifies the rapid implementation of this study, with the aim of improving the care of the population and contributing to a better control of the pandemic.

Trusted Third Party (TTP): e-Health platform

The national number of participants is stored on site in the subject ID log.

The PROMs will be linked by the TTP using the national number with the invoices of medical acts in the IMA database, after approval by the competent chamber of the Information Security Committee.

Participating sites and local investigators

The local investigator, the occupational physician, will collect written informed consent from all participants. In order to facilitate the logistics of participant inclusion, a form to detail participants included will be provided to the investigators. This Subject ID log (appendix) is to be kept by the investigator on site and contains the national number. A copy of the study Subject ID log with the national number and study subject ID is to be provided to the TTP for data linkage.

6.3. RESOURCES

- Purchase, preparation and transport of material for the study will be foreseen by Sciensano.
- Mensura EDPB will organize the research sites through the occupational physician.
- Lab analyses will be performed at Sciensano, ULB and ITG.
- PBMC preparations will be done by Sciensano, ULB and ITG.
- Epidemiological and laboratory data will be analyzed by the Sciensano researchers.
- A budget of 203 600 € was estimated and approved.

7. Risk and benefits for participants

7.1. RISKS

The risks for participants are small, they include side effects of the sampling procedure and vaccination.

- A blood draw can sometimes result in a local hematoma, and rarely in vagal discomfort. Epistaxis can occur in people taking anti-coagulants.
- The most common side effects after BNT162b2 vaccination are usually mild or moderate and resolve within a few days. They include pain and swelling at the injection site, tiredness, headache, muscle and joint pain, chills and fever. They affect about 10% of vaccinated subjects (6).
- Allergic reactions in response to the vaccines do occur, which are severe (anaphylaxis) in very few cases. As for all vaccines, the BNT162b2 vaccine will be given under close supervision with appropriate medical treatment available.

Participants will be clearly informed about these risks, that are minimized due to the expertise of the persons in charge of collecting the samples.

7.2. BENEFITS

In general, the study will provide insight in the possibility of reducing the vaccine dosage required to achieve acceptable immune responses. This might help alleviate the current vaccine scarcity and speed up vaccination campaigns.

In particular, study participants will have the possibility to be vaccinated significantly earlier than foreseen by the Belgian national vaccination campaign, since they all belong to low priority groups. Secondly, those participants receiving a lower dose of the vaccine are expected to experience fewer and less severe side effects than they would with a full dose vaccination (5,9). Finally, if immune responses in the lower dose arm turn out to be inferior to the regular dose arm, these participants will be offered a third, regular dose to boost their immunity.

7.3. CONFIDENTIALITY

At the researcher level, sample results and questionnaires will be pseudonymized via an individual code attributed to each participant. None of the researchers who analyze the data will be involved in participant data collection, nor in the care of COVID-19 participants. Test results will be communicated by the laboratory to the occupational physician of Mensura EDPB or the research nurse by the individual

code attributed to each participant. At all times, the occupational physician and the research nurse are the only ones with access to the identity of the participants.

7.4. BIOLOGICAL SPECIMENS

All the samples, except the PBMCs, collected during the study will be stored in the biobank of Sciensano. Residues remaining after the analyses will be kept for a duration of maximum 10 years if the participant has given consent that the samples can be kept for further research. After 10 years, they will be destroyed. Specimens will be stored in the biobank "Biobank - Sciensano WD12 infectieziekten mens COVID-19" approved by the 'Commissie voor Medische Ethiek UZ Gent' on 17 April 2020 (internal reference: EC 037-2020/mf, appendix 3) with registration number assigned by the FAGG/AFMPS: BB200027.

The PBMC samples will be stored in the Biobank from the laboratory where the preparation took place (Sciensano, ITG or ULB):

- ITM Biobank approved by the 'Commissie voor Medische Ethiek Universitair Ziekenhuis Antwerpen en de Universiteit van Antwerpen" on 8 April 2019 (internal reference: 19/13/168) with registration number assigned by the FAGG/AFMPS: BB190041
- "Biobanque de l'Institut d'Immunologie Médicale (IMI)" approved by the 'le Comité d'Ethique hospital-facultaire Erasme-ULB' on 4 May 2020 with registration number assigned by the FAGG/AFMPS: B2020/002
- Biobank - Sciensano WD12 infectieziekten mens COVID-19" approved by the 'Commissie voor Medische Ethiek UZ Gent' on 17 April 2020 (internal reference: EC 037-2020/mf, appendix 3) with registration number assigned by the FAGG/AFMPS: BB200027.

7.5. INFORMED CONSENT

Information on the study will be provided by Mensura EDPB and informed consent will be obtained from all participants. The informed consent can be found in appendix 1.

7.6. ETHICS COMMITTEE

The study will be conducted in compliance with the principles of the Declaration of Helsinki (2008) and all of the applicable regulatory requirements.

7.7. PROTOCOL AMENDEMENTS

Any substantial change, clarification, or addition to this protocol requires a written protocol amendment, and this must be approved by the Sponsor before the change or addition can be considered effective. In addition, the IEC should be notified and formal approval by the IEC should be obtained as applicable, before the substantial amendment can be implemented. The substantial amendment will reference the protocol by title and version date and must be signed by the principle investigator prior to initiating the change. Once approved, an amendment becomes an integral part of the protocol.

7.8. INSURANCE

During their participation in the clinical investigation the participants will be insured as defined by legal requirements. An insurance with no fault responsibility has been foreseen by the sponsor in accordance with the Belgian law concerning experiments on humans, 7 May 2004.

8. Appendices

8.1. APPENDIX 1: INFORMATION BROCHURE AND INFORMED CONSENT
8.2. APPENDIX 2: QUESTIONNAIRE
8.3. APPENDIX 3: APPROVAL BIOBANK
8.4. APPENDIX 4: SUSAR FORM

9. References

1. Dagan N, Barda N, Kepten E, Miron O, Perchik S, Katz MA, et al. BNT162b2 mRNA Covid-19 Vaccine in a Nationwide Mass Vaccination Setting. N Engl J Med. 2021 Feb 24;

2. Hall VJ, Foulkes S, Saei A, Andrews N, Oguti B, Charlett A, et al. Effectiveness of BNT162b2 mRNA Vaccine Against Infection and COVID-19 Vaccine Coverage in Healthcare Workers in England, Multicentre Prospective Cohort Study (the SIREN Study) [Internet]. Rochester, NY: Social Science Research Network; 2021 Feb [cited 2021 Mar 24]. Report No.: ID 3790399. Available from: https://papers.ssrn.com/abstract=3790399

3. Vasileiou E, Simpson CR, Robertson C, Shi T, Kerr S, Agrawal U, et al. Effectiveness of First Dose of COVID-19 Vaccines Against Hospital Admissions in Scotland: National Prospective Cohort Study of 5.4 Million People [Internet]. Rochester, NY: Social Science Research Network; 2021 Feb [cited 2021 Mar 24]. Report No.: ID 3789264. Available from: https://papers.ssrn.com/abstract=3789264

4. Flamaing J. Vaccination of older persons: a broader perspective. Aging Clin Exp Res. 2009 Oct;21(4–5):372–3; author reply 373-374.

5. Walsh EE, Frenck RW, Falsey AR, Kitchin N, Absalon J, Gurtman A, et al. Safety and Immunogenicity of Two RNA-Based Covid-19 Vaccine Candidates. N Engl J Med. 2020 Dec 17;383(25):2439–50.

6. Polack FP, Thomas SJ, Kitchin N, Absalon J, Gurtman A, Lockhart S, et al. Safety and Efficacy of the BNT162b2 mRNA Covid-19 Vaccine. N Engl J Med. 2020 Dec 31;383(27):2603–15.

7. Earle KA, Ambrosino DM, Fiore-Gartland A, Goldblatt D, Gilbert PB, Siber GR, et al. Evidence for antibody as a protective correlate for COVID-19 vaccines. medRxiv. 2021 Mar 20;2021.03.17.20200246.

8. Baden LR, El Sahly HM, Essink B, Kotloff K, Frey S, Novak R, et al. Efficacy and Safety of the mRNA-1273 SARS-CoV-2 Vaccine. N Engl J Med. 2021 Feb 4;384(5):403–16.

9. Jackson LA, Anderson EJ, Rouphael NG, Roberts PC, Makhene M, Coler RN, et al. An mRNA Vaccine against SARS-CoV-2 — Preliminary Report. N Engl J Med. 2020;12.

10. Tables

Table 1: Timetable of data and sample collection.

Timepoint (days)	Day of dose 1	Day of dose 2	4 weeks post dose 2	6 months post dose 1	1 year post dose 1
Informed consent	X				
Questionnaire	X			X	X
5 ml serum dry tube	X	X	X	X	X
36 ml Heparin tubes*	X		X	X	

* Only for a random selection of 40 study participants, 20 per study arm.
