# Supplementary material for: Safety and immunogenicity of a reduced dose of the BNT162b2 mRNA COVID-19 vaccine (REDU-VAC): A single blind, randomized, non-inferiority trial
Source: PLOS Glob Public Health. 2022 Dec 20;2(12):e0001308. doi: 10.1371/journal.pgph.0001308 (PMC10021431; doi:10.1371/journal.pgph.0001308)
Supplement: S3 Appendix — (PDF) [file pgph.0001308.s010.pdf]

SARS-CoV-2 Specific Binding Antibodies

Enzyme-linked immunosorbent assay

Binding antibodies at baseline and after vaccination were assessed using an enzyme-linked immunosorbent assay (ELISA) for the quantitative detection of IgG-class antibodies to RBD (Receptor Binding Domain, Wuhan strain) (Wantai SARS-CoV-2 IgG ELISA (Quantitative); CE-marked; WS-1396; Beijing Wantai Biological Pharmacy Enterprise Co., Ltd, China). For quantification of antibodies, diluted serum samples (1/10, 1/100, 1/400, 1/1600 and 1/6400) were tested with an internal standard, calibrated against NIBSC 20/136 (First WHO International Standard Anti-SARS-CoV-2 Immunoglobulin), and an external positive control sample included on each plate. Diluted samples were incubated (37°C, 30 min.) with pre-coated micro wells and washed five times. Next, plates were incubated (37°C, 30 min) with horseradish peroxidase (HRP)-conjugated anti-human IgG antibodies and washed five times before adding a TMB and urea peroxide solution for 15 min (37°C, dark). After incubation, a stop solution (0.5 M H2SO4) was added and optical density (OD) was measured at 450 nm using a microplate reader. Net OD values were converted to arbitrary IgG units per ml by interpolation from a point-by-point plot fitted with the standard concentrations and net OD values (correlation coefficient R2≥0.9801), using GraphPad Prism version 9.0.0 for Windows (GraphPad Software, San Diego, California USA) and exported to Microsoft Excel. Antibody measurements were adjusted for any sample dilution, converted to international units per ml (IU/ml) and reported as such. Lower limit of quantification (LLQ) was 5 IU/ml. Clinical performance characteristics of the assay, evaluated in 69 PCR-confirmed COVID-19 patients (comprising mild and severe clinical outcomes, ≥ 15 days post onset of symptoms) and 167 pre-pandemic sera, resulted in a specificity of 100% (95% CI 97,75-100) at a sensitivity of 100% (95% CI 94,73-100) for a cut-off of 6 IU/ml.

Multiplex Immunoassay (Luminex)

Antibody responses at baseline were tested with an in house multiplex immunoassay (MIA). In this test, IgG antibodies to SARS-CoV-2 antigens RBD, S1, S2 and N (Wuhan strain) were measured simultaneously in one assay run. In short, purified antigens RBD (cat n° PX-COV-P046, ProteoGenix, Schiltigheim, France), S1 (cat n° PHA002, Sanyou Biopharmaceuticals, China), S2 (cat n° 40590-V08H1, Sino Biological, China) and N (cat n° PNA006, Sanyou Biopharmaceuticals, China) were coupled covalently to distinct color-coded activated carboxylated beads (Luminex, Austin, Texas, USA). Diluted serum samples (1/100, 1/400, 1/1600 and 1/6400, 1/25600) were measured with the international standard (NIBSC 20/136; first WHO International Standard Anti-SARS-CoV-2 Immunoglobulin), control sera and blanks included on each plate and MFI was converted to IU/mL by interpolation from a five-parameter logistic standard curve.

SARS-CoV-2 Neutralizing Antibodies

Serial dilutions of heat-inactivated serum (1/50-1/25600 in EMEM supplemented with 2mM L-glutamine, 100U/ml - 100µg/ml of Penicillin-Streptomycin and 2% fetal bovine serum) were incubated during 1h (37°C, 7% CO2) with 3xTCID100 of a wild type Wuhan strain (2019-nCoV-Italy-INMI1, reference 008V-03893), the B.1.617.2 Delta variant (83DJ-1) and the BA.1 Omicron variant of SARS-CoV-2, in parallel. Sample-virus mixtures and virus/cell controls were added to Vero cells (18.000 cells/well) in a 96-well plate and incubated for five days (37°C, 7% CO2). The cytopathic effect caused by viral growth was scored microscopically. The Reed-Muench method was used to calculate the neutralizing Ab titer that reduced the number of infected wells by 50% (NT50), which was used as a proxy for the neutralizing Ab concentration in the sample (1–3).

SARS-CoV-2 specific cellular responses

Enzyme-linked immunosorbent spot

Spike-specific cellular responses were tested after vaccination using the Human IFN-γ ELISpot kit (3420-2H) from Mabtech (Stockholm, Sweden) following the recommended protocol. Plates (MAIPSWU10, Mabtech, Stockholm, Sweden) were activated for 15 sec with 50µl of 70% ethanol, and washed with distilled water. Plates were then coated with human IFN-γ antibody (15 µg/ml) overnight at 4°C, washed and blocked with 200µl of Roswell Park Memorial Institute (RPMI) containing 10% fetal bovine serum (FBS) for at least two hours. Next, triplicates of 250 000 PBMC were stimulated in the presence or absence of PepMix SARS-CoV-2 spike glycoprotein peptide pools (SUB1-SUB2, JPT, Berlin, Germany) at 1µg/ml and incubated for 20 hours in a 37°C humidified incubator with 5% CO_2. After incubation, the plates were washed and incubated with the human biotinylated IFN-γ detection antibody (1µg/ml) for 2 hours, washed and the streptavidin–Horseradish Peroxidase (streptavidin-HRP) diluted at 1/750 in PBS-0,5% FBS was added for one hour. 3,3',5,5'-Tetramethylbenzidine substrate was added for minimum 10 min at room temperature. Wells were then washed with distilled water and air-dried. Spot were counted with an ELISpot reader (AID Autoimmun Diagnostika GmbH, Straßberg, Germany), mean values of triplicates were considered for S1 and S2 and expressed per million PBMCs after subtracting the mean of the triplicates of the unstimulated condition. The limit of detection is defined as the mean + 2 SD from naïve subjects at baseline (D0), corresponding to 54 and 66 cells per million PBMCs for S1 and S2, respectively. Data points equal to 0 were attributed the value 1 before transformation.

Flow cytometry

Cells were stimulated in 96-well round-bottom plates with 1 × 10^6 PBMCs in RPMI 1640 medium (Lonza, Basel, Switzerland) supplemented with 10% heat-inactivated FBS (Sigma-Aldrich, Kawasaki, Japan), penicillin/streptomycin, amino acids and PepMix SARS-CoV-2 spike glycoprotein peptide pools (SUB1-SUB2, JPT, Berlin, Germany) in the presence of 1µg/mL purified anti-CD28 antibody (clone CD28.2, BD Biosciences, New Jersey, USA). Both peptide pools were used at 1µg/ml per peptide. Incubation was performed at 37°, 5% CO_2 for 6 hours with 10µg/ml brefeldin A (Sigma-Aldrich, Kawasaki, Japan) added after 90 min. After stimulation, Live/Dead fixable red stain (ThermoFisher, Massachusetts, USA) was used to exclude dead cells and the staining of surface antigens was carried out for 20 min with the following fluorochrome-conjugated antibodies: CD3 BV711 (UCHT-1; BD), anti-CD8 PeCy7 (RPA-T8; BD), CD4 HV450 (RPA-T4; BD). Fixation and permeabilization were performed with Cytofix/Cytoperm (BD) and intracellular staining was carried out for 30 min: IFN-γ FITC (RPA-T8; BD), IL2-APC (MQ1-17H12; BD), TNF-AF700 (Mab11; BD), CD154 APC-Cy7 (TRAP-1 ; BD). Cells stimulated with 1mg/ml Staphylococcus enterotoxin (SEB; Sigma-Aldrich) served as positive controls and unstimulated cells only contained anti-CD28. Samples were acquired on a BD LSRFortessa flow cytometer and analyzed with FlowJo v9. The proportion of cells producing cytokines was determined by subtracting the expression levels/production levels in the unstimulated wells, from the peptide stimulated wells.

References

1. Mariën J, Ceulemans A, Michiels J, Heyndrickx L, Kerkhof K, Foque N, et al. Evaluating SARS-CoV-2 spike and nucleocapsid proteins as targets for antibody detection in severe and mild COVID-19 cases using a Luminex bead-based assay. J Virol Methods. 2021 Feb;288:114025.
2. Selhorst P, Van Ierssel S, Michiels J, Mariën J, Bartholomeeusen K, Dirinck E, et al. Symptomatic SARS-CoV-2 reinfection of a health care worker in a Belgian nosocomial outbreak despite primary neutralizing antibody response. Clin Infect Dis. 2020 Dec 14;ciaa1850.
3. Ariën KK, Heyndrickx L, Michiels J, Vereecken K, Van Lent K, Coppens S, et al. Three doses of BNT162b2 vaccine confer neutralising antibody capacity against the SARS-CoV-2 Omicron variant. NPJ Vaccines. 2022 Mar 8;7(1):35.
